# All-Cause Mortality of Low Birthweight Infants in Infancy, Childhood, and Adolescence: Population Study of England and Wales

**DOI:** 10.1371/journal.pmed.1002018

**Published:** 2016-05-10

**Authors:** W. John Watkins, Sarah J. Kotecha, Sailesh Kotecha

**Affiliations:** Department of Child Health, Cardiff University School of Medicine, Cardiff, United Kingdom; University of Manchester, UNITED KINGDOM

## Abstract

**Background:**

Low birthweight (LBW) is associated with increased mortality in infancy, but its association with mortality in later childhood and adolescence is less clear. We investigated the association between birthweight and all-cause mortality and identified major causes of mortality for different birthweight groups.

**Methods and Findings:**

We conducted a population study of all live births occurring in England and Wales between 1 January 1993 and 31 December 2011. Following exclusions, the 12,355,251 live births were classified by birthweight: 500–1,499 g (very LBW [VLBW], *n =* 139,608), 1,500–2,499 g (LBW, *n =* 759,283), 2,500–3,499 g (*n =* 6,511,411), and ≥3,500 g (*n =* 4,944,949). The association of birthweight group with mortality in infancy (<1 y of age) and childhood/adolescence (1–18 y of age) was quantified, with and without covariates, through hazard ratios using Cox regression. International Classification of Diseases codes identified causes of death. In all, 74,890 (0.61%) individuals died between birth and 18 y of age, with 23% of deaths occurring after infancy. Adjusted hazard ratios for infant deaths were 145 (95% CI 141, 149) and 9.8 (95% CI 9.5, 10.1) for the VLBW and LBW groups, respectively, compared to the ≥3,500 g group. The respective hazard ratios for death occurring at age 1–18 y were 6.6 (95% CI 6.1, 7.1) and 2.9 (95% CI 2.8, 3.1). Male gender, the youngest and oldest maternal age bands, multiple births, and deprivation (Index of Multiple Deprivation score) also contributed to increased deaths in the VLBW and LBW groups in both age ranges. In infancy, perinatal factors, particularly respiratory issues and infections, explained 84% and 31% of deaths in the VLBW and LBW groups, respectively; congenital malformations explained 36% and 23% in the LBW group and ≥2,500 g groups (2,500–3,499 g and ≥3,500 g groups combined), respectively. Central nervous system conditions explained 20% of deaths in childhood/adolescence in the VLBW group, with deaths from neoplasms and external conditions increasingly prevalent in the 1,500–2,499 g and ≥2,500 g birthweight groups. The study would have benefited had we had access to information on gestational age and maternal smoking, but since the former is highly correlated with birthweight and the latter with deprivation, we believe that our findings remain robust despite these shortcomings.

**Conclusions:**

LBW is associated with infant and later child and adolescent mortality, with perinatal factors and congenital malformations explaining many of the deaths. By understanding and ameliorating the influences of upstream exposures such as maternal smoking and deprivation, later mortality can be decreased by reducing the delivery of vulnerable infants with LBW.

## Abstract 2

In a population study, Sailesh Kotecha and colleagues investigate associations between birthweight, all-cause, and cause-specific mortality by birthweight in England and Wales.

## Author Summary

**Why Was This Study Done?:**

- Death rates are increased in babies born with low birthweight (<2,500 g) compared to those born with appropriate birth weight.
- As survival among babies with low birthweight continues to improve, it is important to investigate longer term survival among these children, especially for those born with very low birthweight (<1,500 g) or extremely low birthweight (<1,000 g).

**What Did the Researchers Do and Find?:**

- Using the largest and most recent dataset available, we investigated death rates in children born with low birthweight between birth and 1 y of age and between 1 y and 18 y of age.
- Among 12,355,251 live births in England and Wales between 1 January 1993 and 31 December 2011, there were 74,890 (0.61%) deaths between birth and 18 y of age, with 57,623 (77%) occurring in the first year of life and 17,267 (23%) occurring between 1 and 18 y of age.
- Death rates were higher in babies with low birthweight in both age groups, with death occurring 130 times more frequently in the 500–1,499 g group than the ≥3,500 g group in infancy. The majority (84%) of deaths were due to perinatal events in the very low birthweight group but were more evenly distributed between perinatal events and congenital malformations in the other birthweight groups.
- Deaths in the 1–18 y group were more evenly distributed across causes, with conditions of the nervous system (20%) and respiratory system (16%) being leading causes of death in the lowest birthweight group but cancers and external conditions (including accidents)—both at 20%—being primary causes of death in the ≥2,500 g groups.

**What Do These Findings Mean?:**

- The data showed that low birthweight is associated with increased death rates not only in infancy but also through to adolescence. Since low birthweight has long-term consequences, focusing on upstream events to prevent low birth weight remains an important health goal.

## Introduction

Low birthweight (LBW) (birthweight < 2,500 g) is associated with increased morbidity and mortality in infancy and in adulthood [1–3]. These outcomes have become of greater importance as increasing numbers of extremely LBW and very LBW (VLBW) infants (defined as birthweight < 1,000 g and < 1,500 g, respectively) survive the neonatal period [4,5]. Earlier studies have investigated the relationship between LBW and mortality and morbidity in adulthood [6–8]. Risnes et al. in their systematic review included 15 cohorts with ~40,000 deaths among 400,000 individuals [9]. The cohorts spanned a large range of birth dates and participant ages, although none included children or adolescents. Overall, there was a 6% lower risk of death per kilogramme increase in birthweight. This association was strongest for mortality from cardiovascular disease but weaker for mortality from neoplasms. Class et al., reporting on almost 3.3 million births in Sweden between 1973 and 2007, studied death after the first year of life up to 2012 [10]. They noted 0.73%, 0.44%, 0.35%, and 0.32% mortality in the ≤2,500 g, 2,501–3,000 g, 3,001–3,500 g, and >3,500 g birthweight groups, respectively. Furthermore, LBW was associated with cardiovascular and respiratory disorders, stroke, and type 2 diabetes.

Whilst LBW newborns have increased mortality in the perinatal period and in infancy, it is less clear if there is continuing increased mortality during childhood and adolescence. Due to the increased survival of infants born with extremely LBW (<1,000 g) or VLBW (<1,500 g), longer term outcomes, including morbidity, have become of increasing importance. Our recent data show that both prematurity and fetal growth restriction in term-born infants are associated with later respiratory morbidity [11,12]. However, it is less clear if LBW is associated with increased mortality in childhood and adolescence. Therefore, utilising what we believe to be the largest and most recent cohort to date, we investigated the association between all-cause mortality and birthweight and identified major causes of mortality for different birthweight groups between birth and 18 y of age.

## Methods

The study used anonymised data obtained after formal application to the Office for National Statistics (ONS); thus, the authors did not have access to any identifiable data. Data for the All Wales Perinatal Survey are collected after Confidentiality Advisory Group and ethical approvals. Anonymised data for all live births and deaths up to 18 y of age occurring in England and Wales between 1 January 1993 and 31 December 2011 were available (ONS). The data included birthweight (babies are routinely weighed shortly after birth on calibrated scales) and the covariates gender, Index of Multiple Deprivation (IMD) score, maternal age, and singleton/multiple birth, as these have been shown to be associated with increased mortality [13,14]. In addition, the data included age at time of death and cause of death. ONS data for infant deaths were provided already classified into early neonatal, late neonatal, and post-neonatal deaths; beyond infancy, age in completed years at time of death was provided. Gestational age at birth is not routinely recorded for deaths, but was available for the Welsh data. Welsh data were obtained from the All Wales Perinatal Survey (https://awpsonline.uk/), as a separate cohort for the same time period. International Classification of Diseases (ICD) codes (version 9 up to 1999 and version 10 from 2000 onwards) were used to identify cause of death. Birthweights were classified into four groups: 500–1,499 g (VLBW), 1,500–2,499 g (LBW), 2,500–3,499 g, and ≥3,500 g. The latter two groups were merged for some analyses. The IMD, which is based on a combined measure of deprivation including wealth, schooling, and home ownership in a specific area [15], was divided into quintiles separately for England and Wales then combined into a single variable. There were few missing values for the covariates (maximum of 22,061 [0.2%] for the IMD), and these missing values were unlikely to influence the overall conclusions due to the large dataset.

Cox proportional hazards regression was specified as a means of analysing these data through estimation of hazard ratios and their associated 95% confidence intervals for the birthweight groups for infant mortality (death up to 12 mo of age) and child/adolescent mortality (between 1 and 18 y of age, after censoring deaths occurring in infancy). A Cox model was used to quantify differences between survival rates for the birthweight groups with and without adjustments for relevant covariates. The proportional hazards assumption for the birthweight groups was tested in each model by the addition of an appropriate time-dependant covariate—a product of the system time variable called *T*_ (SPSS notation) and the variable *age*. All covariates were categorical to permit possible non-linear responses. Mortality rates are also given in person-years.

Inspired by peer review, we additionally considered how utilisation of birthweight as a continuous variable might change the results. We used Welsh infant mortality data, and continuous birthweight was expressed as a five-knot restricted cubic spline to provide a linear component within the Cox regression [16]. The knots were chosen in terms of birthweight at 0.5, 1.5, 2.5, 3.5, and 4.5 kg to approximately mimic the categorical birthweight bandings. A five-knot spline has *X* (birthweight) and *X*2, *X*3, and *X*4, which are all functions in *X*. The hazard function within the survival model is then

$$\lambda (t|X)=\lambda (t)\text{exp}(f(X))=\lambda (t)\text{exp}(\beta X+\beta_{2}X2+\beta_{3}X3+\beta_{4}X4)$$

Also following a request at peer review, we utilised the completeness of the data up to age 10 y—i.e., every child in the cohort either turned 10 y or died—to calculate the population attributable fraction (PAF) for deaths among 1 to 10 y olds [17–19].

Several sensitivity analyses were conducted. Since congenital malformations are associated with increased mortality [20], the analyses were repeated after exclusion of deaths from congenital malformations. Because there was an overall decrease in mortality over the last two decades that could influence the results, we also confined the analyses to the most recent 5-y period. In addition, deaths were classified according to the age bands 1–5, 6–10, and 11–18 y. Finally, to assess the potential role of gestation period, we repeated the analyses for the Welsh data with this covariate included, as gestation period was available for all births and deaths for the study period. For the Welsh data, we also compared the infant mortality rate between infants who had intrauterine growth restriction (<10% centile for birthweight adjusted for gender and gestational age) and those with appropriate birthweight for their gestational age (20%–80% centile) using LMSgrowth (Medical Research Council, UK) as the standard [21]. All analyses were performed using PASW 20 (SPSS).

## Results

### All-Cause Mortality

The total cohort included 12,457,528 live births in England and Wales between 1 January 1993 and 31 December 2011. Birthweight data were missing for 92,456 individuals, and data for 9,821 individuals were considered implausible or were outside the limits of analyses, including birthweight of <500 g. The characteristics of the included 12,355,251 individuals, representing 121,208,305 person-years, are shown in Table 1. There were 139,608 (1.1% of all live births), 759,283 (6.1%), 6,511,411 (52.7%), and 4,944,949 (40.0%) live births in the 500–1,499 g, 1,500–2,499 g, 2,500–3,499 g, and ≥3,500 g groups, respectively. In total, 74,890 (0.61%) live births died between birth and 18 y of age, with 77% of deaths occurring in the first 12 mo of life and 23% between 1 and 18 y of age. The mortality rate per 100,000 person-years was 466 for infants and 15.9 for children aged between 1 and 18 y. For the four birthweight groups, there were 25,414 (18.20%), 11,945 (1.57%), 25,750 (0.40%), and 11,781 (0.24%) deaths respectively.

**Table 1:** Mortality rates for birthweight groups, gender, deprivation, maternal age and number of births.

Group	Alive at 18 y		Death up to 18 y (Total)				Died <1 y				Died 1–18 y				Total Live Born
	Number	Percent	Number	Percent	Rate*	RR	Number	Percent	Rate*	RR	Number	Percent	Rate*	RR	
**Total**	12,280,361	99.39%	74,890	0.61%	61.8		57,623	0.47%	466.4		17,267	0.14%	15.9		12,355,251
**Birthweight group**															
500–1,499 g	114,194	81.80%	25,414	18.20%	1,837.9	75.4	24,679	17.68%	1,985.2	129.7	735	0.53%	53.2	5.0	139,608
1,500–2,499 g	747,338	98.43%	11,945	1.57%	160.0	6.6	9,743	1.28%	145.3	9.5	2,202	0.29%	29.5	2.8	759,283
2,500–3,499 g	6,485,661	99.60%	25,750	0.40%	40.2	1.7	16,561	0.25%	28.8	1.9	9,189	0.14%	14.4	1.3	6,511,411
≥3,500 g^#^	4,933,168	99.76%	11,781	0.24%	24.4	1.0	6,640	0.13%	15.3	1.0	5,141	0.10%	10.6	1.0	4,944,949
**Gender**															
Male^#^	6,292,489	99.33%	42,702	0.67%	68.7	1.0	32,993	0.52%	520.8	1.0	9,709	0.15%	17.4	1.0	6,335,191
Female	5,987,872	99.47%	32,188	0.53%	54.5	0.8	24,630	0.41%	409.1	0.8	7,558	0.13%	14.3	0.8	6,020,060
**Deprivation (IMD) quintile**															
Most deprived	3,321,894	99.18%	27,305	0.82%	83.5	1.9	21,165	0.63%	631.9	2.0	6,140	0.18%	20.9	1.7	3,349,199
2	2,622,628	99.37%	16,615	0.63%	65.0	1.5	12,930	0.49%	489.9	1.5	3,685	0.14%	16.1	1.3	2,639,243
3	2,242,871	99.46%	12,258	0.54%	55.5	1.3	9,400	0.42%	416.8	1.3	2,858	0.13%	14.4	1.2	2,255,129
4	2,061,202	99.53%	9,815	0.47%	47.8	1.1	7,440	0.36%	359.2	1.1	2,375	0.11%	12.9	1.1	2,071,017
Least deprived^#^	2,010,161	99.57%	8,649	0.43%	43.1	1.0	6,462	0.32%	320.1	1.0	2,187	0.11%	12.1	1.0	2,018,810
**Maternal age band**															
<16 y	24,461	98.70%	322	1.30%	121.5	2.2	255	1.03%	1,028.9	2.5	67	0.27%	27.9	1.9	24,783
16–20 y	1,186,635	99.12%	10,542	0.88%	88.0	1.6	8,185	0.68%	683.7	1.6	2,357	0.20%	21.9	1.5	1,197,177
21–25 y	2,570,198	99.32%	17,673	0.68%	68.1	1.3	13,446	0.52%	519.6	1.2	4,227	0.16%	18.1	1.2	2,587,871
26–30 y^#^	3,703,449	99.45%	20,668	0.55%	54.0	1.0	15,573	0.42%	418.2	1.0	5,095	0.14%	14.8	1.0	3,724,117
31–35 y	3,231,430	99.49%	16,541	0.51%	53.0	1.0	12,835	0.40%	395.2	0.9	3,706	0.11%	13.2	0.9	3,247,971
36–40 y	1,351,350	99.44%	7,614	0.56%	64.5	1.2	6,076	0.45%	447.1	1.1	1,538	0.11%	14.7	1.0	1,358,964
41–45 y	203,374	99.30%	1,429	0.70%	85.5	1.6	1,172	0.57%	572.3	1.4	257	0.13%	17.5	1.2	204,803
46+ y	9,464	98.98%	98	1.02%	135.5	2.5	78	0.82%	815.7	2.0	20	0.21%	31.9	2.2	9,562
**Number born**															
Singleton^#^	11,930,690	99.44%	67,376	0.56%	57.2	1.0	50,647	0.42%	422.1	1.0	16,729	0.14%	15.8	1.0	11,998,066
Multiple	349,671	97.90%	7,506	2.10%	220.3	3.9	6,968	1.95%	1,950.9	4.6	538	0.15%	17.6	1.1	357,177

*Rate per 100,000 person-years.

^#^Reference value.

RR, rate ratio.

Infant mortality rates were significantly greater in the VLBW and LBW groups: the rate ratio and rate per 100,000 person-years were 129.7 and 1,985.2 for the 500–1,499 g group, 9.5 and 145.3 for the 1,500–2,499 g group, 1.9 and 28.8 for the 2,500–3,499 g group, and 1.0 (reference value) and 15.3 for the ≥3,500 g group, respectively. In addition, boys fared worse than girls (S1 and S2 Figs) even with the exclusion of external conditions (including accidents) as defined by ICD codes. The relative risk ratio of female:male deaths was 0.79 and 0.78 with and without external conditions, respectively, for the <1 y group and 0.82 and 0.87 with and without external conditions, respectively, for the 1–18 y group. Increased deprivation and multiple births were associated with more deaths in all birthweight groups. Maternal age showed a U-shaped relationship, with the greatest mortality in children born to the youngest and oldest mothers (Table 1). The PAF for infant deaths and for deaths between 1 and 10 y of age was 56.6% and 10.7%, respectively, for the comparison between the 500–1,499 g and 1,500–2,499 g groups combined and the ≥2,500 g groups (2,500–3,499 g and ≥3,500 g groups combined), and 51.0% and 3.8%, respectively, when the <1,500 g group and ≥2,500 g groups were compared.

For deaths occurring between 1 and 18 y of age, the rates per 100,000 person-years were 53.2, 29.5, 14.4, and 10.6, respectively, for the 500–1,499 g, 1,500–2,499 g, 2,500–2,499 g, and ≥3,500 g groups. The rate ratios were 5.0, 2.8, and 1.3 for the first three groups, respectively, compared to the ≥3,500 g group. Gender, deprivation, and maternal age bands showed similar patterns as for infant deaths, but the difference between singleton and multiple births was decreased.

**Fig 1:**
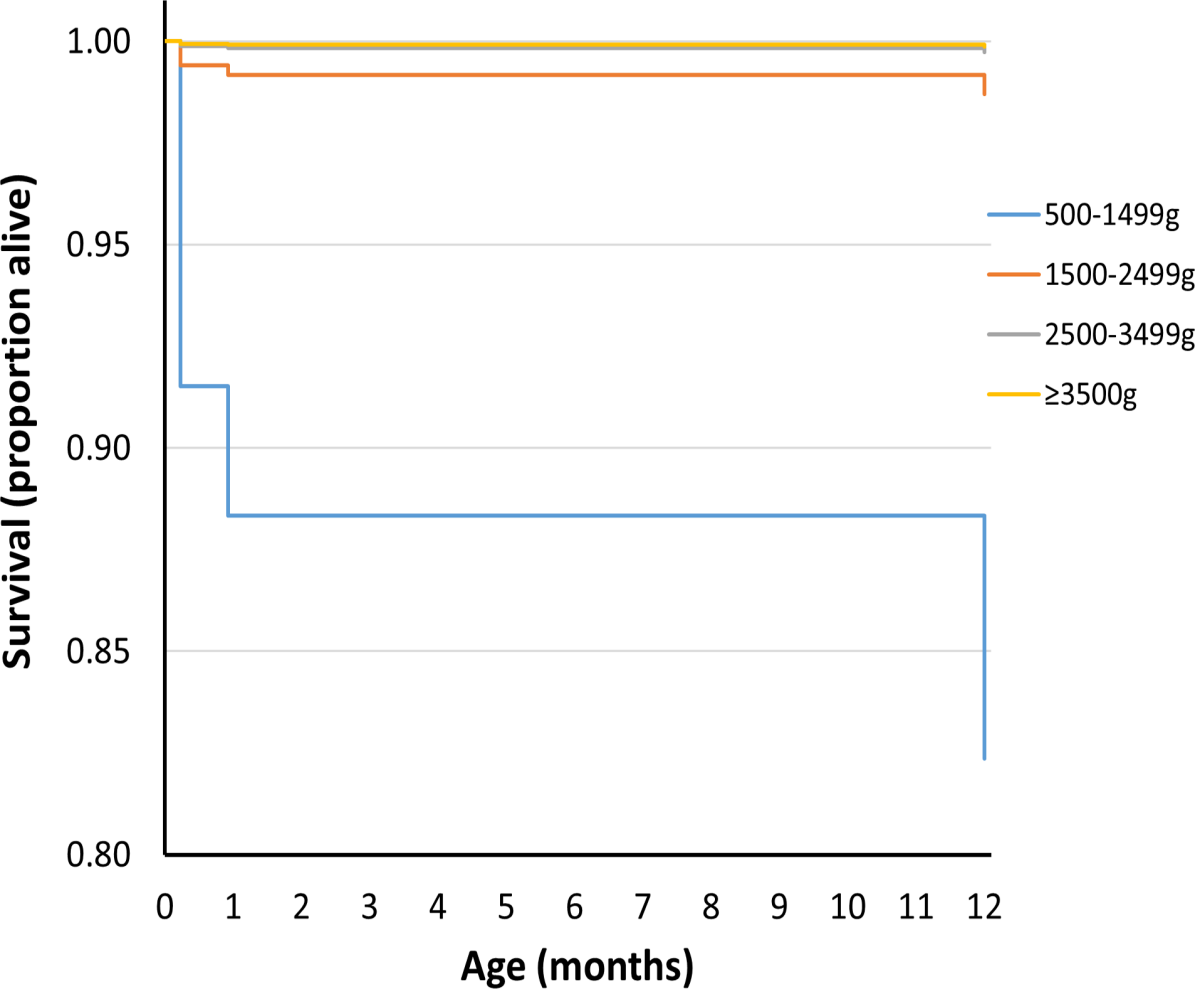
Survival curve by birthweight group for deaths up to 1 y of age for UK population between 1993 and 2011.

**Fig 2:**
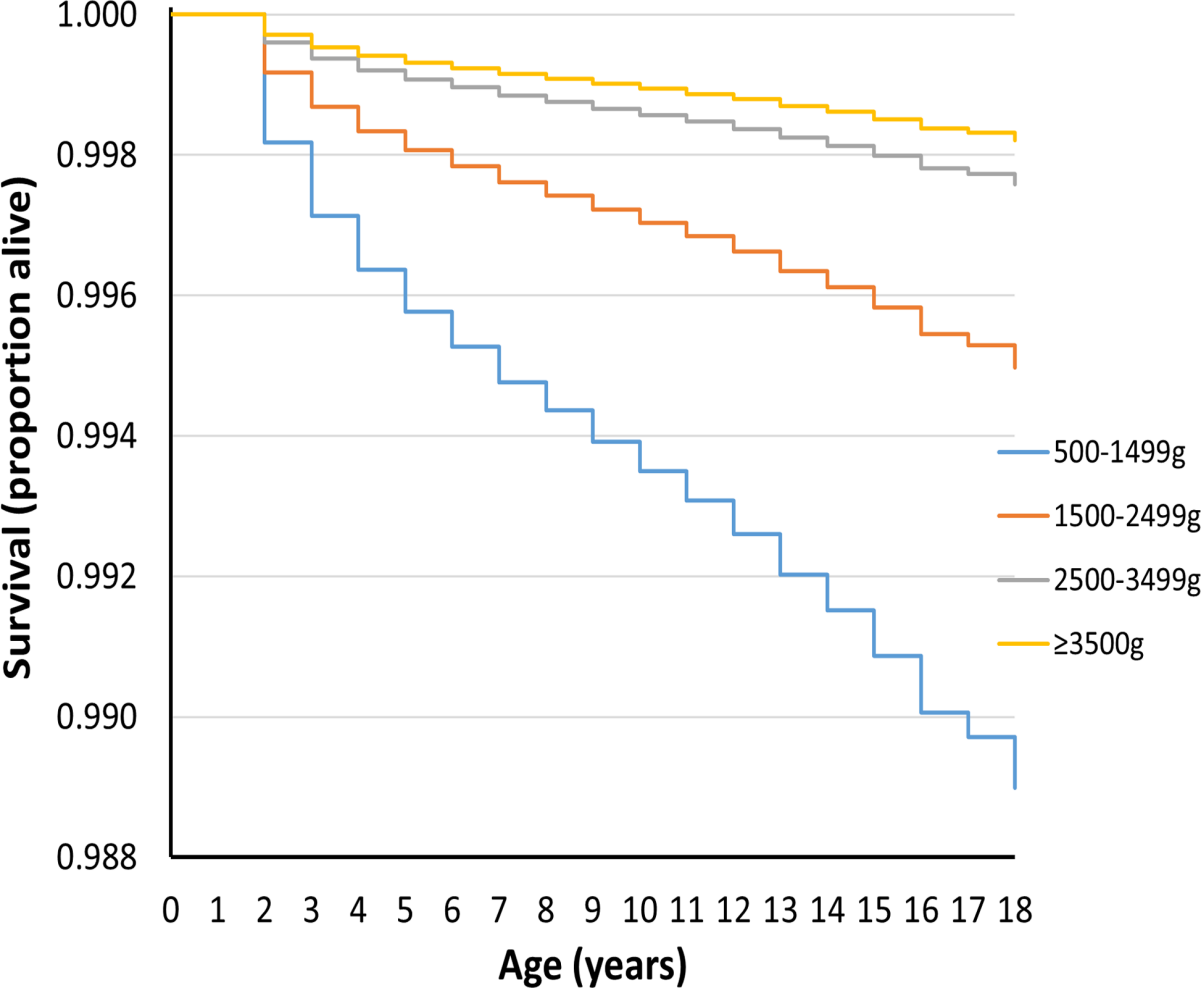
Survival curve by birthweight group for deaths between 1 and 18 y of age for UK population between 1993 and 2011.

Figs 1 and 2 show survival curves for the four birthweight groups in infancy and between 1 and 18 y of age (after censoring of deaths occurring in infancy). In addition to confirming the association of LBW and increased mortality in infancy, the results show that mortality rates were also increased for the lower birthweight groups in childhood/adolescence. Table 2 shows hazard ratios adjusted for deprivation alone and for deprivation, maternal age, multiple birth status, and gender. An inverse relationship was noted between the birthweight groups and mortality, which was largely unaffected after adjustments for covariates.

**Table 2:** Unadjusted and adjusted hazard ratios for death in first year of life and for death between 1 and 18 y of age.

Birthweight Group	Hazard Ratio (95% CI) for Mortality in Infancy			Hazard Ratio (95% CI) for Mortality between 1 and 18 y of Age		
	Unadjusted	Adjusted for Deprivation	Fully Adjusted*	Unadjusted	**Adjusted for Deprivation**	**Fully Adjusted** *
500–1,499 g	141.8 (138.0, 145.7)	136.4 (132.7, 140.2)	145.4 (141.4, 149.4)	6.2 (5.7, 6.7)	5.9 (5.5, 6.4)	6.6 (6.1, 7.1)
1,500–2,499 g	9.6 (9.3, 9.9)	9.2 (8.9, 9.5)	9.8 (9.5, 10.1)	2.8 (2.7, 3.0)	2.7 (2.6, 2.8)	2.9 (2.8, 3.1)
2,500–3,499 g	1.9 (1.8, 1.9)	1.8 (1.8, 1.9)	1.9 (1.8, 1.9)	1.4 (1.3, 1.4)	1.3 (1.3, 1.4)	1.3 (1.3, 1.4)
≥3,500 g (ref)	1	1	1	1	1	1

*Adjusted for deprivation, maternal age, gender, and multiple birth status.

When Welsh infant mortality data were investigated utilising birthweight splines, we utilised the spline function

$$F(X)=\beta X+\beta_{2}X2+\beta_{3}X3+\beta_{4}X4$$

The coefficients β_2_, β_3_, and β_4_ were not significant (S9 Table), which implies that the log of the hazard function λ is basically linear in birth weight (see S3 Fig). The actual expression is

λ(t|X)=λ(t)exp(f(X))=λ(t)exp(−2.370X+0.027X2+0.063X3+0.049X4)

When the hazard ratios were calculated at the midpoints between the knots, the values were very close to those noted following Cox regression of the Welsh data using the categorical definitions (see S10 Table).

### Cause-Specific Mortality

**Fig 3:**
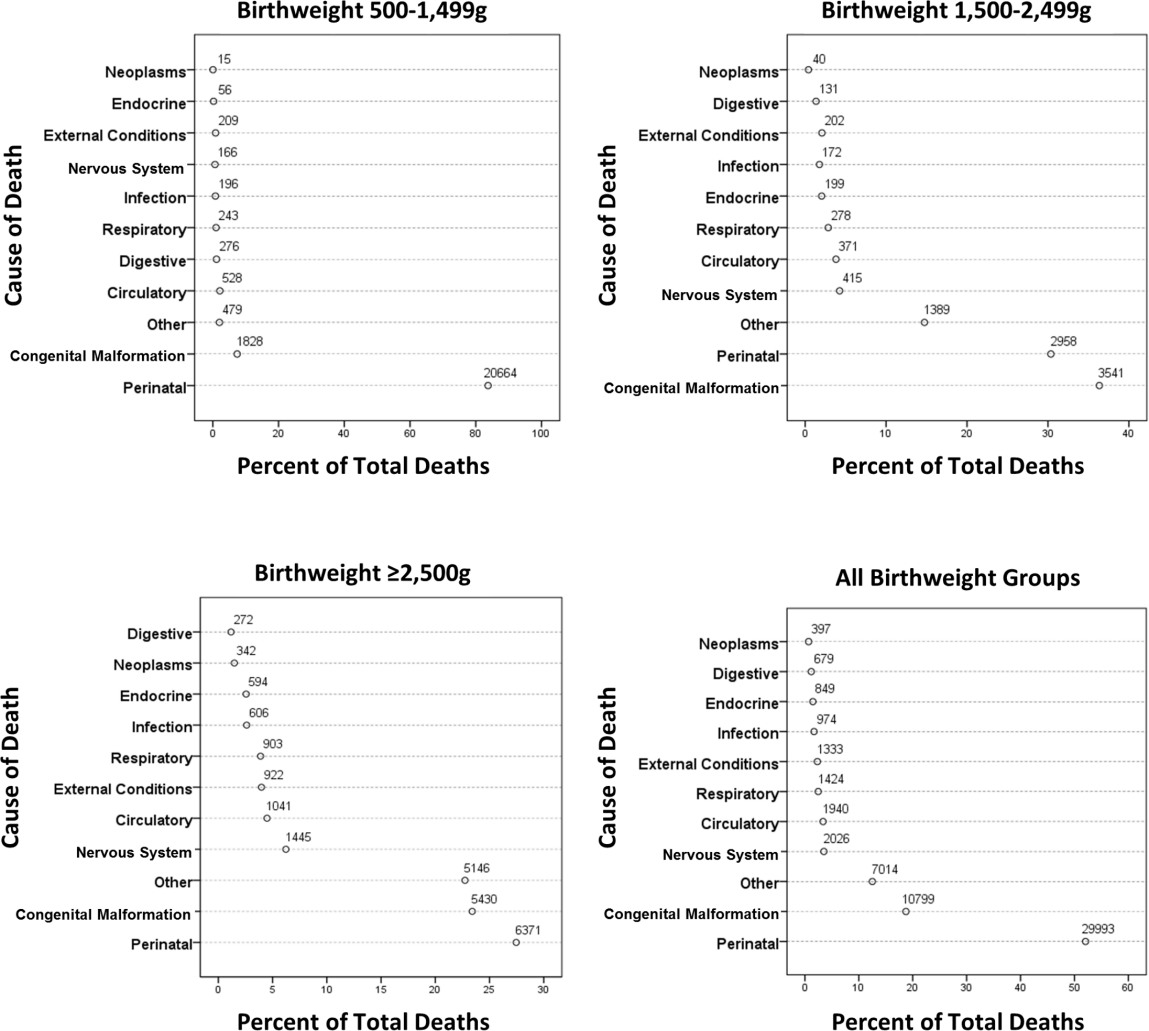
Causes of death in the first year by birthweight group.

Fig 3 shows grouped causes of infant death for each birthweight group, with the ≥2,500 g groups combined. In all, 84% of deaths in the <1,500 g group were related to perinatal events, especially prematurity. For the 1,500–2,499 g group, perinatal events were responsible for 31% of deaths, with congenital anomalies explaining 36% of deaths. Perinatal and congenital anomalies explained approximately half of the deaths in the ≥2,500 g groups. Further breakdown of the most prevalent causes of infant deaths is shown in Table 3. Prematurity and hypoxic conditions explained most of the perinatal deaths, and circulatory conditions explained a large number of deaths from congenital anomalies, but with significant contributions related to respiratory, central nervous system, and chromosomal abnormalities. Conditions related to prematurity within perinatal deaths were 1,890 and 10.4 more likely in the VLBW and LBW groups, respectively, compared to the ≥2,500 g groups. For congenital anomalies, there was a 28-fold greater risk of dying in the <1,500 g group and a nearly 10-fold greater risk in the 1,500–2,499 group compared to the ≥2,500 groups.

**Table 3:** Detailed cause of death for the most prevalent causes for the infant group.

Cause of Death	Measure	Birthweight Group			Total
		500–1,499 g	1,500–2,499 g	≥2,500 g	
**Perinatal deaths**					
Prematurity and LBW	Number	10,929	326	474	11,729
	Rate*	7,828.4	42.9	4.1	94.9
	Rate ratio**	1,890.9	10.4	1.0	
Hypoxia/asphyxia/respiration/pneumonia	Number	5,281	1,297	3,128	9,706
	Rate*	3,782.7	170.8	27.3	78.6
	Rate ratio**	138.6	6.3	1.0	
Infection	Number	1,101	287	519	1,907
	Rate*	788.6	37.8	4.5	15.4
	Rate ratio**	174.1	8.3	1.0	
Other	Number	718	315	754	1,787
	Rate*	514.3	41.5	6.6	14.5
	Rate ratio**	78.2	6.3	1.0	
Digestive	Number	1,368	184	90	1,642
	Rate*	979.9	24.2	0.8	13.3
	Rate ratio**	1,240.4	30.7	1.0	
Haemorrhage	Number	792	111	172	1,075
	Rate*	567.3	14.6	1.5	8.7
	Rate ratio**	378.2	9.8	1.0	
Birth trauma	Number	178	150	672	1,000
	Rate*	127.5	19.8	5.9	8.1
	Rate ratio**	21.7	3.4	1	
Cardiovascular	Number	217	151	381	749
	Rate*	155.4	19.9	3.3	6.1
	Rate ratio**	46.7	6.0	1.0	
Skin	Number	80	137	181	398
	Rate*	57.3	18.0	1.6	3.2
	Rate ratio**	36.3	11.4	1.0	
Total	Number	20,664	2,958	6,371	29,993
	Rate*	14,801.4	389.6	55.6	242.8
	Rate ratio**	266.2	7.0	1.0	
**Congenital malformation**					
Circulatory system	Number	381	891	2,696	3,968
	Rate*	272.9	117.4	23.5	32.1
	Rate ratio**	11.6	5.0	1.0	
Respiration	Number	358	551	596	1,505
	Rate*	256.4	72.6	5.2	12.2
	Rate ratio**	49.3	14.0	1.0	
Chromosomal	Number	294	689	338	1,321
	Rate*	210.6	90.7	3.0	10.7
	Rate ratio**	71.4	30.8	1.0	
Nervous system	Number	291	406	511	1,208
	Rate*	208.4	53.5	4.5	9.8
	Rate ratio**	46.7	12.0	1.0	
Musculoskeletal system	Number	147	356	612	1,115
	Rate*	105.3	46.9	5.3	9.0
	Rate ratio**	19.7	8.8	1.0	
Other	Number	190	373	401	964
	Rate*	136.1	49.1	3.5	7.8
	Rate ratio**	38.9	14.0	1.0	
Urinary system	Number	72	181	125	378
	Rate*	51.6	23.8	1.1	3.1
	Rate ratio**	47.3	21.9	1.0	
Digestive system	Number	95	94	151	340
	Rate*	68.1	12.4	1.3	2.8
	Rate ratio**	51.6	9.4	1.0	
Total	Number	1,828	3,541	5,430	10,799
	Rate*	1,309.4	466.4	47.4	87.4
	Rate ratio**	27.6	9.8	1.0	

Total person-years for first year of life is 12,355,251.

*Rate per 100,000 person-years.

**Rate compared to the ≥2,500 g groups.

**Fig 4:**
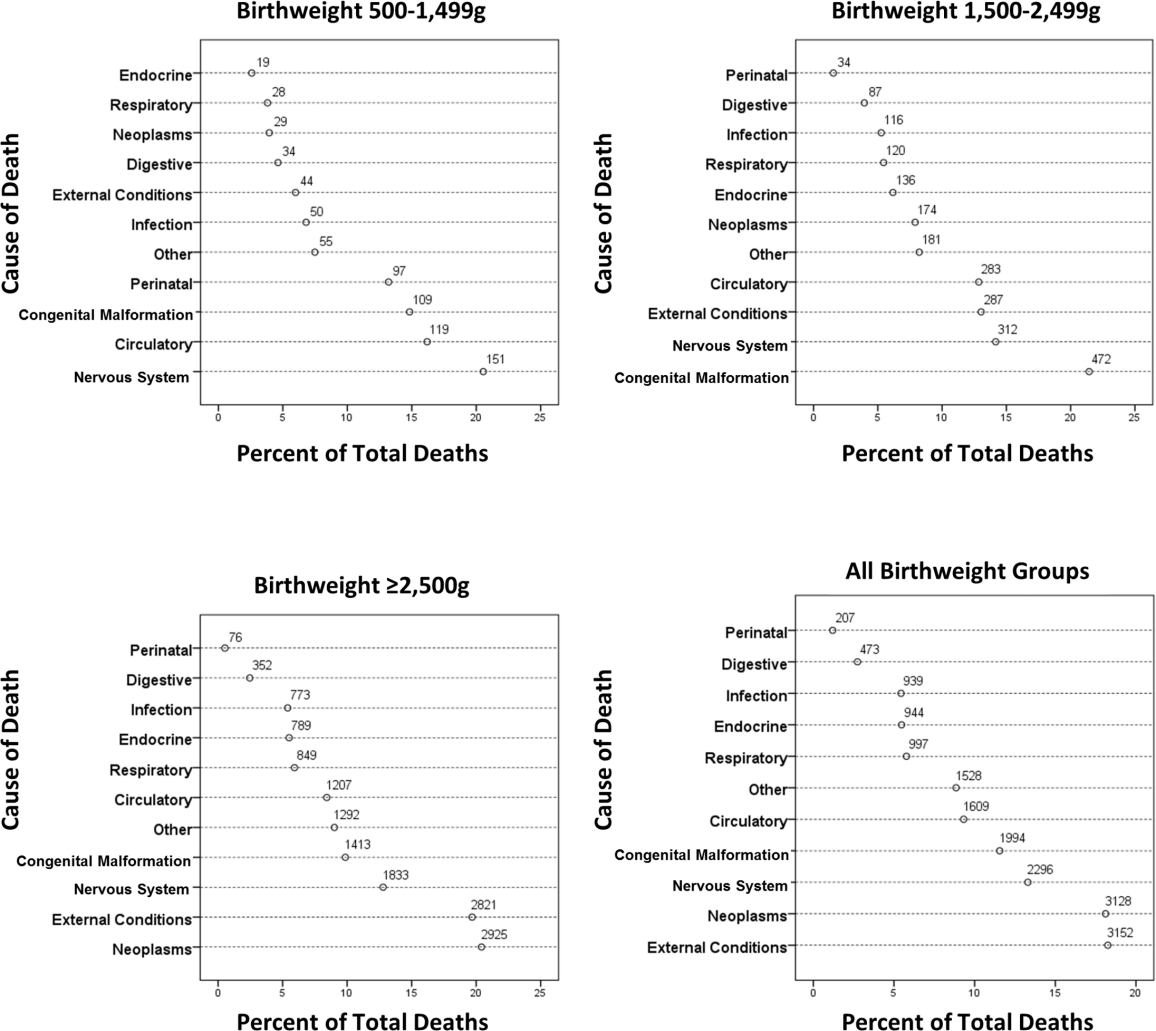
Causes of death between 1 and 18 y of age by birthweight group.

Fig 4 shows grouped causes of death between 1 and 18 y by birthweight group. The causes of death were more diverse, but perinatal causes remained significant in the <1,500 g group and congenital anomalies in the 500–1,499 g and 1,500–2,499 g groups, but less so for the ≥2,500 g groups. Further breakdown of the most prevalent causes of death is shown in Table 4, where conditions associated with the nervous system, particularly cerebral palsy, were prevalent, with a rate ratio for cerebral palsy of 16.5 and 4.3 for the VLBW and LBW groups, respectively, compared to the ≥2,500 g groups. Overall, individuals in the VLBW and LBW groups were 6.3 and 5.0 times more likely to die from congenital anomalies than individuals in the ≥2,500 g groups. Respiratory conditions were responsible for more deaths in the two lower birthweight groups, particularly from influenza/pneumonia, where the rate ratios were 8.4 and 3.7, respectively, compared to the ≥2,500 g groups. Neoplasms were the most common cause of death in the ≥2,500 g groups, explaining 20% of deaths. External conditions including accidents explained 18% of deaths for all birthweight groups; however, an increasing prevalence was observed with increasing birthweight, i.e., 6% of deaths in the 500–1,499 g group increasing to almost 20% in the ≥2,500 g group (Fig 4).

**Table 4:** Detailed cause of death for the most prevalent causes for the 1–18 y age group.

Cause of Death	Measure	Birthweight Group			Total
		500–1,499 g	1,500–2,499 g	≥2,500 g	
**Nervous system**					
Cerebral palsy and associated conditions	Number	82	115	401	598
	Rate*	6.6	1.7	0.4	0.6
	Rate ratio**	16.5	4.3	1.0	
Other	Number	32	84	421	537
	Rate*	2.6	1.3	0.4	0.5
	Rate ratio**	6.1	3.0	1.0	
Episodic disorders	Number	10	39	339	388
	Rate*	0.8	0.6	0.3	0.4
	Rate ratio**	2.6	1.7	1.0	
Inflammatory diseases	Number	6	16	254	276
	Rate*	0.5	0.2	0.3	0.3
	Rate ratio**	1.9	1.0	1.0	
Hereditary and degenerative diseases	Number	17	40	296	353
	Rate*	1.4	0.6	0.3	0.3
	Rate ratio**	4.7	2.1	1.0	
Diseases of myoneural junction and muscle	Number	4	18	122	144
	Rate*	0.3	0.3	0.1	0.1
	Rate ratio**	2.7	2.3	1.0	
Total	Number	151	312	1,833	2,296
	Rate*	12.2	4.7	1.8	2.1
	Rate ratio**	6.7	2.6	1.0	
**Congenital malformation**					
Circulatory system	Number	35	169	631	835
	Rate*	2.8	2.5	0.6	0.8
	Rate ratio**	4.5	4.0	1.0	
Nervous system	Number	25	98	302	425
	Rate*	2.0	1.5	0.3	0.4
	Rate ratio**	6.7	4.9	1.0	
Other	Number	42	122	372	536
	Rate*	3.4	1.8	0.4	0.5
	Rate ratio**	9.1	4.9	1.0	
Chromosomal	Number	7	83	108	198
	Rate*	0.6	1.2	0.1	0.2
	Rate ratio**	5.1	11.3	1.0	
Total	Number	109	472	1,413	1,994
	Rate*	8.8	7.0	1.4	1.8
	Rate ratio**	6.3	5.0	1.0	
**Neoplasms**					
Lymphoid, thyroid, and other endocrine	Number	6	79	1,080	1,165
	Rate*	0.5	1.2	1.1	1.1
	Rate ratio**	0.5	1.1	1.0	
Central nervous system	Number	8	31	856	895
	Rate*	0.6	0.5	0.9	0.8
	Rate ratio**	0.8	0.5	1.0	
Bone	Number	2	5	142	149
	Rate*	0.2	0.1	0.1	0.1
	Rate ratio**	1.1	0.5	1.0	
Mesothelial and other soft tissue	Number	2	11	126	139
	Rate*	0.2	0.2	0.1	0.1
	Rate ratio**	1.3	1.3	1.0	
Other	Number	11	46	619	676
	Rate*	0.9	0.7	0.6	0.6
	Rate ratio**	1.4	1.1	1.0	
Urinary	Number	0	2	102	104
	Rate*	0.0	0.0	0.1	0.1
	Rate ratio**	0.0	0.3	1.0	
Total	Number	29	174	2,925	3,128
	Rate*	2.3	2.6	2.9	2.9
	Rate ratio**	0.8	0.9	1.0	
**Respiratory**					
Influenza and pneumonia	Number	52	125	500	677
	Rate*	4.2	1.9	0.5	0.6
	Rate ratio**	8.4	3.7	1.0	
Other	Number	67	158	707	932
	Rate*	5.4	2.4	0.7	0.9
	Rate ratio**	7.7	3.4	1.0	
Total	Number	119	283	1,207	1,609
	Rate*	9.6	4.2	1.2	1.5
	Rate ratio**	8.0	3.5	1.0	

Total person-years for age 1 to 18 y is 108,853,054.

*Rate per 100,000 person-years.

**Rate compared to the ≥2,500 g groups.

### Sensitivity Analyses

#### Congenital malformations

Results were only slightly different when congenital malformations were removed (S1 and S2 Tables).

#### Gestational age

Gestational age was available for all Welsh live births via the All Wales Perinatal Survey (https://awpsonline.uk/). For the study period, there were 635,428 live births and 3,836 deaths between birth and 18 y of age for individuals with birthweight above 500 g. For the VLBW group, the median gestation period was 29 wk (10th and 90th percentiles at 25 and 33 wk). Corresponding data for the LBW, 2,500–3,499 g, and ≥3,500 g groups were 36 (32, 39), 39 (37, 41), and 40 (39, 41) wk, respectively. S3 Table shows the Cox regression results for the two age groups, which were very similar to the overall results. For all gestational age groups, there was an increased risk of death in infancy if the infant had intrauterine growth restriction, compared to appropriately grown infants (S4 Table), although the difference was not significant for the 23–28 wk gestation group.

#### Temporal effects on mortality

The time-varying covariate was significant in all regression models, suggesting that hazard ratios may be temporally affected. The effect of adjustment for the time-varying covariate only slightly increased the hazard ratios (S5 Table). When the data were classified into shorter time periods (1993–1996, 1997–2001, 2002–2006, and 2007–2011), the results remained essentially unchanged, with small increases for each time period (S6 and S7 Tables). When deaths at age 1–18 y were divided into age groups, younger groups had greater mortality (hazard ratio of 8.0 for 1–5 y olds, 4.2 for 6–10 y olds, and 2.7 for 11–18 y olds) (S8 Table).

## Discussion

This population-based study included data from 12,457,528 live births occurring in England and Wales between 1993 and 2011. Overall, 0.61% (*n =* 74,890) died, with 23% of all deaths occurring at 1–18 y of age. Mortality was greater in the two lower birthweight groups than in the two heavier birthweight groups in infancy and between 1–18 y of age, with little modification by covariates. Adjusted hazard ratios for death in infancy were 145 (95% CI 141, 149) and 9.8 (95% CI 9.5, 10.1) for the VLBW and LBW groups, respectively, compared to the ≥3,500 g group, and 6.6 (95% CI 6.1, 7.1) and 2.9 (95% CI 2.8, 3.1) for these birthweight groups for deaths at 1–18 y of age. Gender, maternal age, multiple births, and deprivation also contributed to increased deaths in the two lower birthweight groups. Deaths related to perinatal factors and congenital malformations, especially from cardiovascular causes, were predominant in the two lower birthweight groups in infancy and later in childhood and adolescence.

Increased infant mortality rate has been reported previously for lower birthweight groups [9,22–26]. Horbar et al. reported an infant death rate of 12.4% for the 501–1,500 g birthweight group—compared to 17.7% for the 500–1,499 g group our study—for 669 American hospitals in 2009; the data are not directly comparable as they defined infant mortality as death occurring prior to hospital discharge [27]. Doyle et al. reported that 66.9% of live-born individuals with a birthweight of 500–999 g survived to 2 y of age excluding those with congenital anomalies; when we calculated the same outcome for our data, the result was remarkably similar, at 63.1% [4]. Mortality after infancy in our <2,500 g groups is lower than Class and colleagues’ report of 0.73% mortality in their ≤2,500 g group, most likely due to their follow-up to up to 34 y of age [10]. Of note is the systematic review by Risnes et al., who reported a 6% lower risk of death for every kilogramme increase in birthweight [9].

Power and Li, using a cohort of ~17,000 individuals born in 1958 in Britain, showed an inverse relationship between birthweight and infant mortality but not for deaths occurring >1 y of age [22]. Friedlander et al., in a larger cohort of 92,408 births in Jerusalem between 1964 and 1976, noted a strong relationship between LBW and death before 1 y of age but not between LBW and death at 1–15 y of age [23]. Horta et al. studied 5,914 Brazilian children born in 1982 for over 20 y, noting a significant relationship between LBW and mortality up to 4 y of age but not beyond [28]. Kajantie et al. studied 13,830 individuals born between 1924 and 1944 in Finland, following them from 1971 to a mean age of 56 y. They noted a positive relationship between LBW and all-cause mortality at all ages for women but not for men, for whom only infant mortality was associated with LBW [24]. Many of these studies were constrained by the limited numbers of deaths that occurred, especially beyond infancy.

Furthermore, many of these studies were conducted on populations several decades ago, focusing on the consequences of LBW for adulthood morbidity and mortality. Overall survival rates have improved, especially for infants born with birthweight ≤ 1,500 g, and, more recently, for those with birthweight ≤ 1,000 g; our data, up to 2011, are more recent. By reporting mortality rate data as both hazard ratios and person-years, we have taken into account that children born recently will not have reached the age of 18 y during the study period. Our data show that mortality is increased markedly in the lower birthweight groups compared to the heavier birthweight groups beyond infancy. Even in sensitivity analyses, the conclusions remain robust. Males fared worse in both infancy and beyond. The mechanisms for this gender difference remain unclear. Deprivation, multiple births, and maternal age have previously been shown to be associated with increased mortality in infants, but we confirm their importance for increased mortality also in childhood and adolescence [29–32].

Perinatal causes accounted for 52% of all deaths in infancy but accounted for 84% of deaths in the VLBW group. Perhaps unsurprisingly, prematurity predominated as the cause of death in this group, but of note is the fact that hypoxic/anoxic causes were responsible for a significant number of deaths. Whilst there have been great improvements in maternal and neonatal outcomes over the last two decades, these data suggest that targeting upstream risk factors for LBW remains an important health goal. Congenital malformations were also important causes of infant death, especially in the LBW group and the ≥2,500 g groups, explaining 36% and 23% of deaths, respectively. Whilst congenital heart defects were responsible for a large number of the deaths among those who died due to congenital malformation, disorders of the respiratory and central nervous system and chromosomal afflictions resulted in significant mortality.

For the 1–18 y age group, the causes of death were more diverse, although perinatal causes (precise causes were not recorded) were responsible for 13% of deaths in the VLBW group, and congenital malformations for 10%–21% of deaths in all birthweight groups. Neoplasms and accidents (external conditions) were important causes of death in the ≥2,500 g groups. This concurs with the findings presented in Syddall et al., who noted an increase in neoplasms with increasing birthweight, although their cohort covered a much wider age group (up to age 88 y) than ours [33]. Similar data were also reported by Sidebotham et al. [34,35]. Rates of respiratory causes of death were similar between the groups. Hazard ratios were greater for almost all causes of death (except neoplasms and external causes) in the VLBW and LBW groups compared to the ≥2,500 g groups, thus suggesting that LBW has consequences well beyond early childhood and that preventing LBW remains an important heath target. Whilst our aim was to establish whether there is continuing mortality after low weight at birth and, if so, the underlying causes for the increased mortality, it is clearly important to establish which upstream casual factors such as tobacco smoke and deprivation lead to delivery of LBW infants. Medical factors may also contribute to earlier delivery, resulting in LBW infants; thus, caution is required not to interpret LBW as being necessarily on the causal pathway to later mortality. Furthermore, additional information is also required, as many deaths in the VLBW and LBW groups were reported as being due to prematurity. In our study, maternal age, multiple births, and deprivation all appeared to be associated with increased later mortality associated with LBW, thus providing additional interventional targets to improve mortality related to LBW. Although we did not have information on antenatal maternal smoking exposure, it has been shown to be associated with LBW and clearly provides another target for intervention [36]. In addition, infant deaths were increased in growth-restricted infants compared to appropriately grown infants (S4 Table). Clearly, any factor—including maternal, fetal, and placental factors and also medical interventions—leading to growth-restricted infants at birth is associated with adverse longer term outcomes and provides an opportunity to investigate the upstream casual factors resulting in LBW.

Crump et al. concluded from analyses of 674,820 births in Sweden from 1973 to 1979 that cause-specific mortality was significantly related to gestational age at birth in their 1–5 y age group but not for older age groups, although the latter conclusion may be due to limited numbers of deaths [37]. It has been suggested that relating mortality outcomes to gestational age may be preferable to relating them to birthweight as gestational age reflects the maturity of the infant at birth. Whilst this may be true, we believe that our results are equally valid, especially as birthweight was highly correlated with gestational age, as shown by analyses of the Welsh births, for which both gestational age and birthweight were available. The analyses of these 639,294 live births robustly supported our overall conclusions. Importantly, by investigating the association of birthweight with mortality, we have identified three important areas that need special attention: preterm labour, anoxic/hypoxic conditions of the newborn, and congenital malformations. As major congenital malformations are associated with increased mortality, we conducted sensitivity analyses excluding individuals with these conditions, but the relationship between lower birthweight and mortality remained unchanged.

Survival rates improved over the 19-y study period; thus, we conducted another sensitivity analysis investigating death up to 5 y of age for the most recent 5-y period. The association of VLBW and LBW with later mortality remained, thus suggesting that the overall findings remain robust. We also report the PAF for both infant and post-infant mortality, showing that this fraction, unsurprisingly, is greatest in infancy, but for infants born at <2,500 g, it is nearly 11% for deaths occurring between 1 and 10 y of age. This implies that 11% of deaths between 1 and 10 y of age could be prevented by eliminating LBW.

The major strengths of this study and the reason why it is a relevant addition to the literature in this area are the inclusion of >12 million births and 74,890 deaths and the availability of relevant and recent data up to the age of 18 y. We were also able to identify the underlying causes of death. We had few missing data for the covariates we included, but we did not have data on gestational age or maternal smoking. Since the former is highly correlated with birthweight and the latter with deprivation, we believe that our findings remain robust despite these shortcomings. Additionally, by also treating birthweight as a continuous variable for the Welsh data, we were able to show that there was no disadvantage in using banded, as opposed to continuous, birthweight data.

In conclusion, using what we believe to be the largest and most recent population-based cohort that has been used to investigate this area, we have shown that VLBW and LBW are associated with mortality in infancy and in childhood/adolescence. Major causes of death include perinatal and congenital malformations in infancy, which continue to explain many deaths that occur up to 18 y of age. LBW is clearly associated with later mortality in childhood and adolescence. By understanding and ameliorating the influences of upstream exposures such maternal smoking and deprivation, later mortality can be decreased by reducing the delivery of vulnerable infants with LBW.

## Supporting Information

S1 Fig Association of gender with survival up to 1 y of age for UK population between 1993 and 2011. (TIF)

S2 Fig Association of gender with survival between 1 and 18 y for UK population between 1993 and 2011. (TIF)

S3 Fig Plot of spline function against birthweight following Cox regression for infant death on continuous Welsh birthweight data. (DOCX)

S1 Table Unadjusted and adjusted hazard ratios for death in the first year of life with deaths due to congenital malformations excluded. (DOCX)

S2 Table Unadjusted and adjusted hazard ratios for death between 1 and 18 y of age with deaths due to congenital malformations excluded. (DOCX)

S3 Table Hazard ratios for deaths in the Wales study population between 1993 and 2011 for the four birthweight groups. (DOCX)

S4 Table Comparison of infant deaths in appropriately grown and growth-restricted infants. (DOCX)

S5 Table Hazard ratios for infant death and death between 1 and 18 y of age for 1993–2011 with adjustment for a time-varying covariate. (DOCX)

S6 Table Hazard ratios for death before 1 y for the four birthweight groups for 1993–2011 split into four time periods. (DOCX)

S7 Table Hazard ratios for death between 1 and 18 y of age for the four birthweight groups for 1993–2011 split into four time periods. (DOCX)

S8 Table Mortality rates in different childhood age groups. (DOCX)

S9 Table Spline coefficients following Cox regression for infant death on continuous Welsh birthweight data. (DOCX)

S10 Table Spline coefficients following Cox regression for infant death on continuous Welsh birthweight data. (DOCX)

S1 STROBE Checklist Checklist of items that should be included in reports of observational studies. (DOC)

## Abbreviations

ICD: International Classification of Diseases
IMD: Index of Multiple Deprivation
LBW: low birthweight
ONS: Office for National Statistics
PAF: population attributable fraction
VLBW: very low birthweight
