# Correction: All-Cause Mortality of Low Birthweight Infants in Infancy, Childhood, and Adolescence: Population Study of England and Wales

**DOI:** 10.1371/journal.pmed.1002069

**Published:** 2016-06-15

**Authors:** The PLOS Medicine Staff

## Notice of Republication

This article was republished on May 19^th^, 2016 to correct poor figure quality in the PDF version, which was introduced during the typesetting process. The publisher apologizes for these issues with the figure quality. Please download this article again to view the updated version.
