# Supplementary figures and images for: All-Cause Mortality of Low Birthweight Infants in Infancy, Childhood, and Adolescence: Population Study of England and Wales
Source: PLoS Med. 2016 May 10;13(5):e1002018. doi: 10.1371/journal.pmed.1002018 (PMC4862683; doi:10.1371/journal.pmed.1002018)

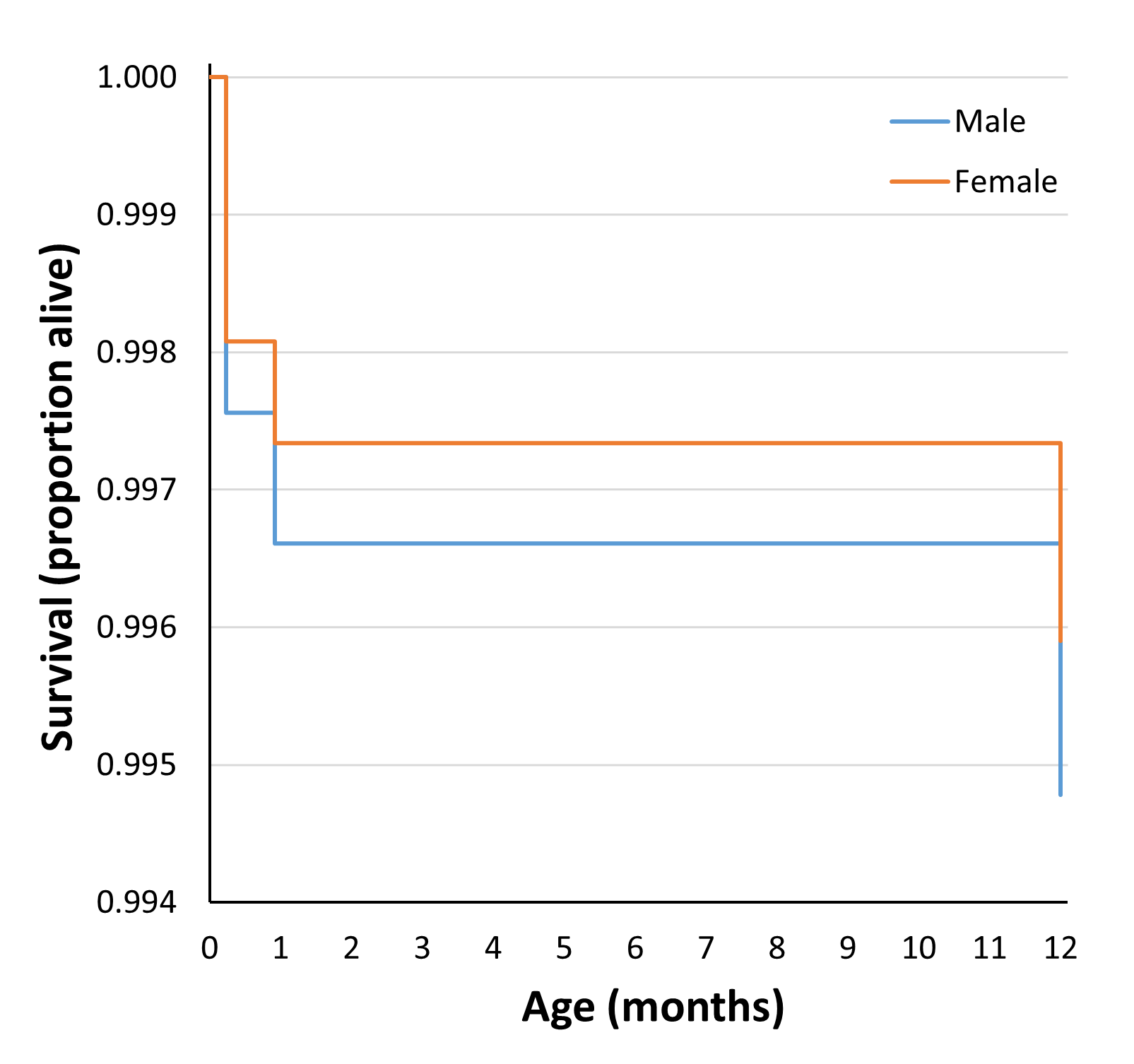

Supplement: S1 Fig — (TIF) [file pmed.1002018.s001.tif]

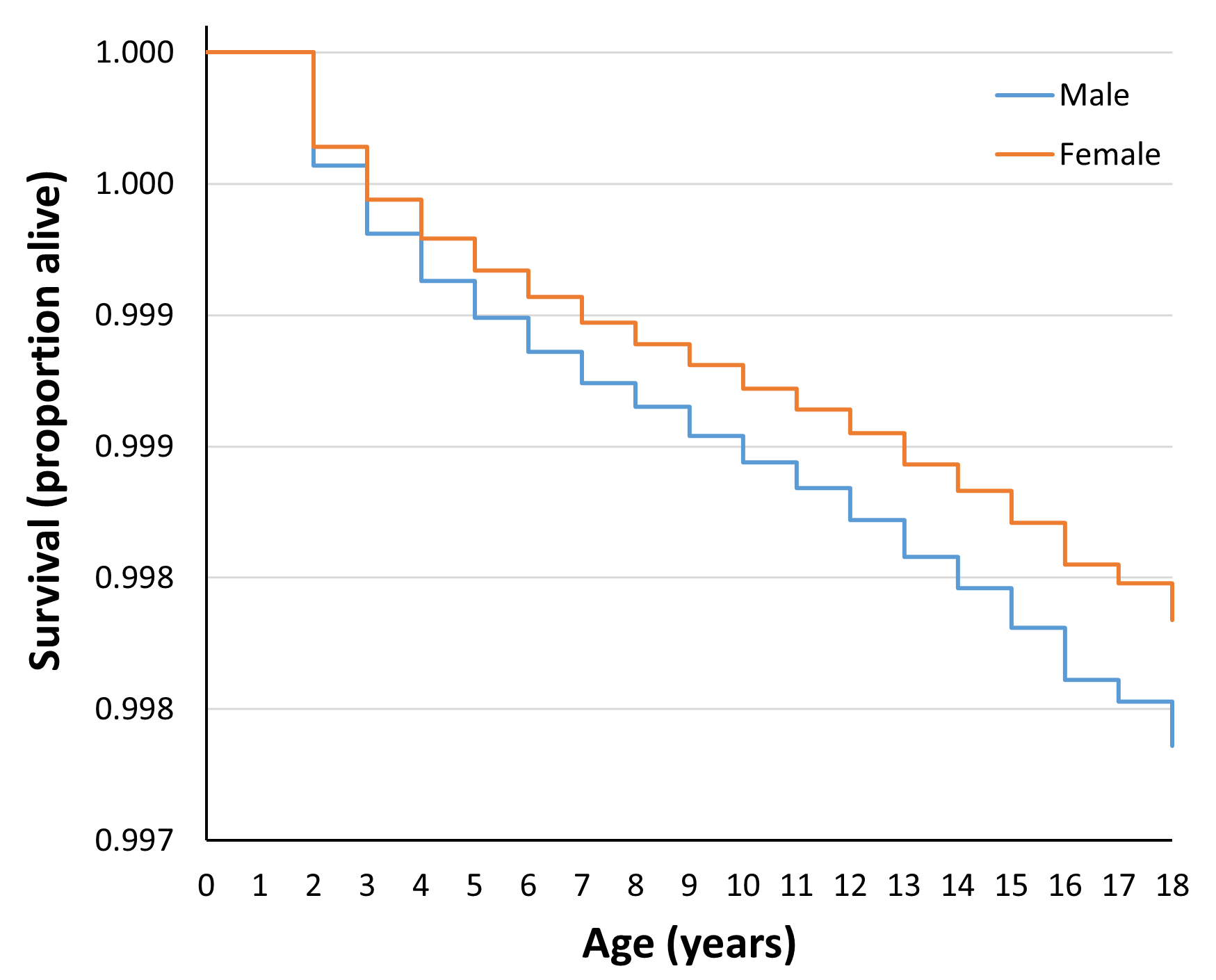

Supplement: S2 Fig — (TIF) [file pmed.1002018.s002.tif]

**S3 Fig:**


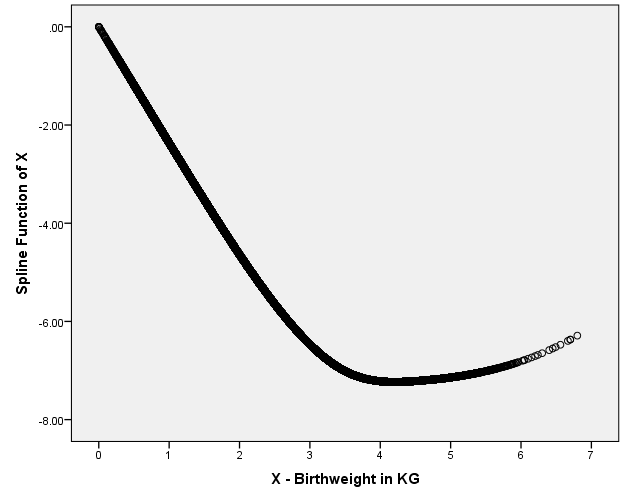

Supplement: S3 Fig — (DOCX) [file pmed.1002018.s003.docx]
