# Supplementary material for: All-Cause Mortality of Low Birthweight Infants in Infancy, Childhood, and Adolescence: Population Study of England and Wales
Source: PLoS Med. 2016 May 10;13(5):e1002018. doi: 10.1371/journal.pmed.1002018 (PMC4862683; doi:10.1371/journal.pmed.1002018)
Supplement: S1 Table — (DOCX) [file pmed.1002018.s004.docx]

**S1 Table. Unadjusted and adjusted hazard ratios for death in the first year of life with deaths due to congenital malformations excluded**.

|  |  | | |
| --- | --- | --- | --- |
| **Birthweight Group** | **Unadjusted** | **Adjusted for deprivation** | **Fully Adjusted*** |
| **500-1,499g** | 160.7 (156.1,165.5) | 154.7 (150.1, 159.3) | 163.1 (158.2, 168.1) |
| **1,500-2,499g** | 7.4 (7.2,7.7) | 7.1 (6.9, 7.4) | 7.5 (7.3, 7.8) |
| **2,500-3,499g** | 1.7 (1.6,1.8) | 1.7 (1.6, 1.7) | 1.7 (1.7, 1.8) |
| $\boldsymbol{\geq}$**3,500g (ref)** | 1 | 1 | 1 |

* adjusted for deprivation, maternal age, gender and multiple birth status.
