# Supplementary material for: All-Cause Mortality of Low Birthweight Infants in Infancy, Childhood, and Adolescence: Population Study of England and Wales
Source: PLoS Med. 2016 May 10;13(5):e1002018. doi: 10.1371/journal.pmed.1002018 (PMC4862683; doi:10.1371/journal.pmed.1002018)
Supplement: S2 Table — (DOCX) [file pmed.1002018.s005.docx]

**S2 Table. Unadjusted and adjusted hazard ratios for death between 1 and 18 y of age with deaths due to congenital malformations excluded.**

|  |  | | |
| --- | --- | --- | --- |
| **Birthweight Group** | **Unadjusted** | **Adjusted for deprivation** | **Fully Adjusted*** |
| **500-1,499g** | 5.6 (5.1, 6.1) | 5.4 (4.9, 5.8) | 5.9 (5.4,6.4) |
| **1,500-2,499g** | 2.4 (2.2, 2.5) | 2.2 (2.1, 2.4) | 2.4 (2.3, 2.6) |
| **2,500-3,499g** | 1.3 (1.2, 1.3) | 1.2, (1.2, 1.3) | 1.3 (1.2, 1.3) |
| $\boldsymbol{\geq}$**3,500g (ref)** | 1 | 1 | 1 |

* adjusted for deprivation, maternal age, gender and multiple birth status.
