# Supplementary material for: All-Cause Mortality of Low Birthweight Infants in Infancy, Childhood, and Adolescence: Population Study of England and Wales
Source: PLoS Med. 2016 May 10;13(5):e1002018. doi: 10.1371/journal.pmed.1002018 (PMC4862683; doi:10.1371/journal.pmed.1002018)
Supplement: S3 Table — (DOCX) [file pmed.1002018.s006.docx]

**S3 Table. Hazard ratios for deaths in the Wales study population between 1993 and 2011 for the four birthweight groups.**

| **Birthweight Group** | **death before 1 year** |  | **death between 1 and 18 years of age** |
| --- | --- | --- | --- |
| **500-1,499g** | 142.1 (126.5, 159.6) |  | 6.1 (4.3, 8.6) |
| **1,500-2,499g** | 9.0 (7.8, 10.3) |  | 2.9 (2.4, 3.7) |
| **2,500-3,499g** | 1.8 (1.6, 2.1) |  | 1.2 (1.1, 1.4) |
| $\boldsymbol{\geq}$**3,500g (ref)** | 1 |  | 1 |
