# Supplementary material for: All-Cause Mortality of Low Birthweight Infants in Infancy, Childhood, and Adolescence: Population Study of England and Wales
Source: PLoS Med. 2016 May 10;13(5):e1002018. doi: 10.1371/journal.pmed.1002018 (PMC4862683; doi:10.1371/journal.pmed.1002018)
Supplement: S4 Table — (DOCX) [file pmed.1002018.s007.docx]

**S4 Table. Comparison of infant deaths in appropriately grown and growth-restricted infants.**

| **23-28 weeks** |  | **AGA** | **%** |  | **IUGR** | **%** | **Odds ratio (95%CI) for death in IUGR and AGA groups** |
| --- | --- | --- | --- | --- | --- | --- | --- |
|  | **Alive** | 1,443 | 68.81 |  | 272 | 65.39 | 1.2 |
|  | **Died** | 654 | 31.19 |  | 144 | 34.62 | (0.9,1.5) p=0.095 |
|  | **Total** | 2,097 |  |  | 416 |  |  |
| **29-33 weeks** | **Alive** | 5,895 | 97.20 |  | 1,428 | 92.25 | 2.9 |
|  | **Died** | 170 | 2.80 |  | 120 | 7.75 | (2.3,3.7) p< 0.001 |
|  | **Total** | 6,065 |  |  | 1,548 |  |  |
| **34-36 weeks** | **Alive** | 17,276 | 99.24 |  | 3,307 | 97.52 | 3.3 |
|  | **Died** | 133 | 0.76 |  | 84 | 2.48 | (2.5, 4.4) p<0.001 |
|  | **Total** | 17,409 |  |  | 3,391 |  |  |
| **Term** | **Alive** | 360,333 | 99.83 |  | 49,945 | 99.43 | 3.4 |
|  | **Dead** | 615 | 0.17 |  | 288 | 0.57 | (2.9,3.9) p<0.001 |
|  | **Total** | 360,948 |  |  | 50,233 |  |  |
