# Supplementary material for: All-Cause Mortality of Low Birthweight Infants in Infancy, Childhood, and Adolescence: Population Study of England and Wales
Source: PLoS Med. 2016 May 10;13(5):e1002018. doi: 10.1371/journal.pmed.1002018 (PMC4862683; doi:10.1371/journal.pmed.1002018)
Supplement: S5 Table — (DOCX) [file pmed.1002018.s008.docx]

**S5 Table. Hazard ratios for infant death and death between 1 and 18 y of age for 1993–2011 with adjustment for a time-varying covariates.**

| **Birthweight Group** | **Infant death** | | **Death 1-18** | |
| --- | --- | --- | --- | --- |
|  | **Hazard ratio** | **p** | **Hazard ratio** | **p** |
| **500-1,499g** | 280.3 (270.3, 290.8) | <0.001 | 8.6 (6.1, 12.1) | <0.001 |
| **1,500-2,499g** | 16.3 (15.7, 16.9) | <0.001 | 3.6 (2.9, 4.6) | <0.001 |
| **2,500-3,499g** | 2.6 (2.5, 2.6) | <0.001 | 1.5 (1.3, 1.7) | <0.001 |
| $\boldsymbol{\geq}$**3,500g (ref)** | 1 |  | 1 | 1 |
| **T_COV** | 1.72 (1.69,1.75) | <0.001 | 1.02 (1.01, 1.04) | <0.001 |
