# Supplementary material for: All-Cause Mortality of Low Birthweight Infants in Infancy, Childhood, and Adolescence: Population Study of England and Wales
Source: PLoS Med. 2016 May 10;13(5):e1002018. doi: 10.1371/journal.pmed.1002018 (PMC4862683; doi:10.1371/journal.pmed.1002018)
Supplement: S6 Table — (DOCX) [file pmed.1002018.s009.docx]

**S6 Table. Hazard ratios for death before 1 y for the four birthweight groups for 1993–2011 split into four time periods.**

| **Birthweight Group** | **1993-1996** | **1997-2001** | **2002-2006** | **2007-2011** |
| --- | --- | --- | --- | --- |
| **500-1,499g** | 128.6 (122.1,135.4) | 146.5 (138.9,154.5) | 146.3 (138.2,154.8) | 148.6 (140.1,157.1) |
| **1,500-2,499g** | 8.4 (7.9,8.9) | 9.7 (9.2,10.4) | 10.2 (9.6,10.9) | 10.2 (9.6,10.8) |
| **2,500-3,499g** | 1.7 (1.6,1.7) | 2.0 (1.9,2.1) | 2.1 (1.9,2.1) | 1.9 (1.8,2.0) |
| $\boldsymbol{\geq}$**3,500g (ref)** | 1 | 1 | 1 | 1 |
