# Supplementary material for: All-Cause Mortality of Low Birthweight Infants in Infancy, Childhood, and Adolescence: Population Study of England and Wales
Source: PLoS Med. 2016 May 10;13(5):e1002018. doi: 10.1371/journal.pmed.1002018 (PMC4862683; doi:10.1371/journal.pmed.1002018)
Supplement: S7 Table — (DOCX) [file pmed.1002018.s010.docx]

**S7 Table. Hazard ratios for death between 1 and 18 y of age for the four birthweight groups for 1993–2011 split into four time periods.**

| **Birthweight Group** | **1993-1996** | **1997-2001** | **2002-2006** | **2007-2011** |
| --- | --- | --- | --- | --- |
| **500-1,499g** | 8.2 (6.7,9.9) | 7.1 (5.9,8.5) | 9.0 (7.6, 10.7) | 10.5 (8.5,12.9) |
| **1,500-2,499g** | 3.1 (2.7,3.6) | 3.6 (3.2,4.0) | 3.6 (3.1,4.0) | 4.3 (3.7,4.9) |
| **2,500-3,499g** | 1.5 (1.4,1.6) | 1.4 (1.3,1.5) | 1.4 (1.3, 1.6) | 1.6 (1.4,1.8) |
| $\boldsymbol{\geq}$**3,500g (ref)** | 1 | 1 | 1 | 1 |
