# Supplementary material for: All-Cause Mortality of Low Birthweight Infants in Infancy, Childhood, and Adolescence: Population Study of England and Wales
Source: PLoS Med. 2016 May 10;13(5):e1002018. doi: 10.1371/journal.pmed.1002018 (PMC4862683; doi:10.1371/journal.pmed.1002018)
Supplement: S8 Table — (DOCX) [file pmed.1002018.s011.docx]

**S8 Table. Mortality rates in different childhood age groups.**

|  | **Hazard Ratio plus CI for Death after first year** | | |
| --- | --- | --- | --- |
|  | **Death aged 1-18 years** | | |
| **Birthweight Group** | **Unadjusted** | **Adjusted for deprivation** | **Fully Adjusted*** |
| **500-1,499g** | 6.2 (5.7, 6.7) | 5.9 (5.5, 6.4) | 6.6 (6.1,7.1) |
| **1,500-2,499g** | 2.8 (2.7, 3.0) | 2.7 (2.6, 2.8) | 2.9 (2.8, 3.1) |
| **2,500-3,499g** | 1.4 (1.3, 1.4) | 1.3, (1.3, 1.4) | 1.3 (1.3, 1.4) |
| $\boldsymbol{\geq}$**3,500g (ref)** | 1 | 1 | 1 |
|  | | | |
|  | **Death aged 1-5 years** | | |
| **Birthweight Group** | **Unadjusted** | **Adjusted for deprivation** | **Fully Adjusted*** |
| **500-1,499g** | 8.0 (7.3, 8.7) | 7.6 (6.9, 8.3) | 8.4 (7.7, 9.2) |
| **1,500-2,499g** | 3.4 (3.2, 3.6) | 3.2 (3.0, 3.4) | 3.5 (3.3, 3.7) |
| **2,500-3,499g** | 1.4 (1.4, 1.5) | 1.4 (1.3, 1.4) | 1.4 (1.3, 1.5) |
| $\boldsymbol{\geq}$**3,500g (ref)** | 1 | 1 | 1 |
|  | | | |
|  | **Death aged 6-10 years** | | |
| **Birthweight Group** | **Unadjusted** | **Adjusted for deprivation** | **Fully Adjusted*** |
| **500-1,499g** | 4.2 (3.4, 5.1) | 4.0 (3.2, 4.9) | 4.5 (3.6, 5.5) |
| **1,500-2,499g** | 2.3 (2.0, 2.6) | 2.2 (2.0, 2.5) | 2.4 (2.2, 2.8) |
| **2,500-3,499g** | 1.3 (1.2, 1.4) | 1.3, (1.2, 1.4) | 1.3 (1.2, 1.4) |
| $\boldsymbol{\geq}$**3,500g (ref)** | 1 | 1 | 1 |
|  | | | |
|  | **Death aged 11-18 years** | | |
| **Birthweight Group** | **Unadjusted** | **Adjusted for deprivation** | **Fully Adjusted*** |
| **500-1,499g** | 2.7 (2.1, 3.5) | 2.6 (2.0, 3.4) | 2.8 (2.2, 3.7) |
| **1,500-2,499g** | 1.6 (1.4, 1.9) | 1.6 (1.4, 1.8) | 1.7 (1.5, 2.0) |
| **2,500-3,499g** | 1.2 (1.1, 1.3) | 1.1 (1.0, 1.2) | 1.2 (1.1, 1.3) |
| $\boldsymbol{\geq}$**3,500g (ref)** | 1 | 1 | 1 |

* adjusted for deprivation, maternal age, gender and multiple birth status.
