# Supplementary material for: All-Cause Mortality of Low Birthweight Infants in Infancy, Childhood, and Adolescence: Population Study of England and Wales
Source: PLoS Med. 2016 May 10;13(5):e1002018. doi: 10.1371/journal.pmed.1002018 (PMC4862683; doi:10.1371/journal.pmed.1002018)
Supplement: S9 Table — (DOCX) [file pmed.1002018.s012.docx]

**S9 Table. Spline coefficients following Cox regression for infant death on continuous Welsh birthweight data.**

| Spline variable | Coefficient | Significance (p value) | Hazard ratio= exp(B) | ci low | ci high |
| --- | --- | --- | --- | --- | --- |
| X | -2.37 | 0 | 0.093 | 0.081 | 0.108 |
| X2 | 0.027 | 0.323 | 1.027 | 0.974 | 1.084 |
| X3 | 0.063 | 0.443 | 1.065 | 0.906 | 1.253 |
| X4 | 0.049 | 0.65 | 1.05 | 0.85 | 1.298 |
