# Supplementary material for: All-Cause Mortality of Low Birthweight Infants in Infancy, Childhood, and Adolescence: Population Study of England and Wales
Source: PLoS Med. 2016 May 10;13(5):e1002018. doi: 10.1371/journal.pmed.1002018 (PMC4862683; doi:10.1371/journal.pmed.1002018)
Supplement: S10 Table — (DOCX) [file pmed.1002018.s013.docx]

**S10 Table. Spline coefficients following Cox regression for infant death on continuous Welsh birthweight data.**

| **Birthweight** | **Hazard ratios from COX regression**  **with categorical variables.** | **Approximate Hazard ratios for the midpoints between the knots following COX regression** |
| --- | --- | --- |
| **500-1499g** | 135.1 | 130.7 |
| **1500-2499g** | 9.9 | 13.4 |
| **2500-3499g** | 2.0 | 2.1 |
| **3500-6999g (Ref)** | 1 | 1 |
