# Supplementary material for: All-Cause Mortality of Low Birthweight Infants in Infancy, Childhood, and Adolescence: Population Study of England and Wales
Source: PLoS Med. 2016 May 10;13(5):e1002018. doi: 10.1371/journal.pmed.1002018 (PMC4862683; doi:10.1371/journal.pmed.1002018)
Supplement: S1 STROBE Checklist — (DOC) [file pmed.1002018.s014.doc]

STROBE Statement—checklist of items that should be included in reports of observational studies

|  | | Item No | Recommendation |
| --- | --- | --- | --- |
| **Title and abstract** | | 1 | (*a*) Indicate the study’s design with a commonly used term in the title or the abstract  *The title on page 1 in the main manuscript states the study design*  ***“ALL-CAUSE MORTALITY OF LOW BIRTHWEIGHT INFANTS IN INFANCY, CHILDHOOD AND ADOLESCENCE: POPULATION STUDY OF ENGLAND AND WALES”*** |
|  |  |  | (*b*) Provide in the abstract an informative and balanced summary of what was done and what was found *The Abstract in the main manuscript provides a balanced summary of what was done and what was found.*  **“*Abstract***  ***Background:*** *Low birthweight (LBW) is associated with increased mortality in infancy but its association with mortality in later childhood and adolescence is less clear. We investigated the association between birthweight and all-cause mortality; and identified major causes of mortality for different birthweight groups.*  ***Methods and Findings:*** *Population study of all live-births occurring in England and Wales between 1993 and 2011. Following exclusions, the 12,355,251 live births, with birthweights were classified into very low birthweight, VLBW (500-1499g, n=139,608), LBW (1,500-2499g, n=759,283), 2,500-3499g (n=6,511,411) and ≥3,500g (n=4,944,949) groups. The association of each birthweight group with mortality in infancy (<1 year of age) and between 1-18 years of age was quantified, with and without covariates, through hazard ratios using Cox regression. International Classification of Diseases codes identified causes of death. 74,890 (0.61%) died between birth and 18 years of age with 23% of deaths occurring after infancy. Adjusted hazard ratios for infant deaths were 145 (95% confidence intervals: 141, 149) and 9.8 (9.5, 10.1) for VLBW and LBW groups respectively when compared to the ≥3500g group. The respective hazard ratios for death occurring at age 1-18 years were 6.6 (6.1, 7.1) and 2.9 (2.8, 3.1). Gender (males worse), maternal age (highest for the youngest and oldest bands), multiple births and deprivation also contributed to increased deaths in the lower birthweight groups at both age ranges. In infancy, perinatal factors, particularly respiratory issues and infections, explained 84% and 31% of deaths in the VLBW and LBW groups respectively; congenital malformations explained 36% and 23% in the LBW and ≥2,500g groups respectively. Central nervous system conditions explained 20% of deaths in childhood/adolescence in the VLBW group but deaths from neoplasms and external conditions were associated with higher birthweight. The study would have benefited had we access to gestational age and maternal smoking, but since the former is highly correlated with birthweight and the latter with deprivation, we believe that our findings remain robust despite these short-comings.*  ***Conclusion:*** *Low birthweight is associated with infant and later child and adolescent mortality with perinatal and congenital malformation explaining many of the deaths. By understanding and ameliorating influences of upstream exposures such maternal smoking and deprivation, later mortality can be decreased by reducing the delivery of vulnerable infants with low birthweight.”* |
| Introduction | | | |
| Background/rationale | | 2 | Explain the scientific background and rationale for the investigation being reported  *In the introduction second paragraph in the main manuscript a scientific background and rationale is provided*  *“Whilst mortality of low birthweight newborns is increased in the perinatal period and in infancy, it is less clear if there is continuing mortality during childhood or adolescence. Due to the increased survival of infants born with extremely (<1,000g) or very low (<1,500g) birthweight, longer term outcomes including morbidity have become of increasing importance. Our recent data have shown that both prematurity and fetal growth restriction in term-born infants are associated with later respiratory morbidity. However, it is less clear if low birthweight is associated with increased mortality in childhood and adolescence. We, therefore, utilising what we believe to be the largest and most recent cohort to date investigated the association between all-cause mortality and birthweight; and identified major causes of mortality for different birthweight groups between birth and 18 years of age.”* |
| Objectives | | 3 | State specific objectives, including any prespecified hypotheses  *The objectives are stated in the introduction second paragraph of the main manuscript.*  *“ We, therefore, utilising what we believe to be the largest and most recent cohort to date investigated the association between all-cause mortality and birthweight; and identified major causes of mortality for different birthweight groups between birth and 18 years of age.”* |
| Methods | | | |
| Study design | | 4 | Present key elements of study design early in the paper  *The key elements are presented in the method section first paragraph of the main manuscript*  *“Anonymised data for all live-births and deaths up to 18 years of age occurring in England and Wales between 1993 and 2011 were available (Office for National Statistics, ONS). The data included birthweight (babies are routinely weighed shortly after birth on calibrated scales) and the covariates gender, local deprivation score, maternal age and maternal parity as these have been shown to be associated with increased mortality . In addition, the data included age and cause of each death. ONS data for infant deaths were provided already classified into early, late and post-neonatal deaths; beyond infancy, age in completed years at time of death were provided. Gestational age at birth is not routinely recorded for the deaths except for the Welsh data. Welsh data, was obtained from the All Wales Perinatal Survey (*[*https://awpsonline.uk/*](https://awpsonline.uk/)*), as a separate cohort for the same time period. International Classification of Diseases (ICD) code (version 9 up to 1999 and version 10 from 2000/1 onwards) were used to identify causes of death. Birthweights were classified into four groups: 500-1,499g (VLBW), 1,500-2499g (LBW), 2,500-3499g and ≥3,500g. The latter two groups were merged for some analyses. The index of multiple deprivation (IMD), which is based on a combined measure of deprivation including wealth, schooling, home ownership in a specific area,* *was divided into quintiles separately for England and Wales then combined into a single variable. Although there were few missing values for the covariates (maximum of 22,061 (0.2%) for IMD), these were unlikely to influence the overall conclusions due to the large dataset.”* |
| Setting | | 5 | Describe the setting, locations, and relevant dates, including periods of recruitment, exposure, follow-up, and data collection  *The setting, locations and relevant dates are presented in the method section first paragraph of the main manuscript.*  *“Anonymised data for all live-births and deaths up to 18 years of age occurring in England and Wales between 1993 and 2011 were available (Office for National Statistics, ONS). The data included birthweight (babies are routinely weighed shortly after birth on calibrated scales) and the covariates gender, local deprivation score, maternal age and maternal parity as these have been shown to be associated with increased mortality . In addition, the data included age and cause of each death. ONS data for infant deaths were provided already classified into early, late and post-neonatal deaths; beyond infancy, age in completed years at time of death were provided. Gestational age at birth is not routinely recorded for the deaths except for the Welsh data. Welsh data, was obtained from the All Wales Perinatal Survey (*[*https://awpsonline.uk/*](https://awpsonline.uk/)*), as a separate cohort for the same time period. International Classification of Diseases (ICD) code (version 9 up to 1999 and version 10 from 2000/1 onwards) were used to identify causes of death.”* |
| Participants | | 6 | (*a*) *Cohort study*—Give the eligibility criteria, and the sources and methods of selection of participants. Describe methods of follow-up  *Case-control study*—Give the eligibility criteria, and the sources and methods of case ascertainment and control selection. Give the rationale for the choice of cases and controls  *Cross-sectional study*—Give the eligibility criteria, and the sources and methods of selection of participants |
|  |  |  | (*b*) *Cohort study*—For matched studies, give matching criteria and number of exposed and unexposed  *Case-control study*—For matched studies, give matching criteria and the number of controls per case  *The participants are described in the methods section first paragraph of the main manuscript*  ***“****Anonymised data for all live-births and deaths up to 18 years of age occurring in England and Wales between 1993 and 2011 were available (Office for National Statistics, ONS). The data included birthweight (babies are routinely weighed shortly after birth on calibrated scales) and the covariates gender, local deprivation score, maternal age and maternal parity as these have been shown to be associated with increased mortality . In addition, the data included age and cause of each death. “* |
| Variables | | 7 | Clearly define all outcomes, exposures, predictors, potential confounders, and effect modifiers. Give diagnostic criteria, if applicable  *The variables are defined in the methods section first paragraph of the main manuscript*  *“The data included birthweight (babies are routinely weighed shortly after birth on calibrated scales) and the covariates gender, local deprivation score, maternal age and maternal parity as these have been shown to be associated with increased mortality . In addition, the data included age and cause of each death. ONS data for infant deaths were provided already classified into early, late and post-neonatal deaths; beyond infancy, age in completed years at time of death were provided. Gestational age at birth is not routinely recorded for the deaths except for the Welsh data. Welsh data, was obtained from the All Wales Perinatal Survey (*[*https://awpsonline.uk/*](https://awpsonline.uk/)*), as a separate cohort for the same time period. International Classification of Diseases (ICD) code (version 9 up to 1999 and version 10 from 2000/1 onwards) were used to identify causes of death. Birthweights were classified into four groups: 500-1,499g (VLBW), 1,500-2499g (LBW), 2,500-3499g and ≥3,500g. The latter two groups were merged for some analyses. The index of multiple deprivation (IMD), which is based on a combined measure of deprivation including wealth, schooling, home ownership in a specific area,* *was divided into quintiles separately for England and Wales then combined into a single variable. Although there were few missing values for the covariates (maximum of 22,061 (0.2%) for IMD), these were unlikely to influence the overall conclusions due to the large dataset.”* |
| Data sources/ measurement | | 8* | For each variable of interest, give sources of data and details of methods of assessment (measurement). Describe comparability of assessment methods if there is more than one group  *Data sources and details of methods of assessment are given in the methods first paragraph.*  ***“****Anonymised data for all live-births and deaths up to 18 years of age occurring in England and Wales between 1993 and 2011 were available (Office for National Statistics, ONS). The data included birthweight (babies are routinely weighed shortly after birth on calibrated scales) and the covariates gender, local deprivation score, maternal age and maternal parity as these have been shown to be associated with increased mortality . In addition, the data included age and cause of each death. ONS data for infant deaths were provided already classified into early, late and post-neonatal deaths; beyond infancy, age in completed years at time of death were provided. Gestational age at birth is not routinely recorded for the deaths except for the Welsh data. Welsh data, was obtained from the All Wales Perinatal Survey (*[*https://awpsonline.uk/*](https://awpsonline.uk/)*), as a separate cohort for the same time period. International Classification of Diseases (ICD) code (version 9 up to 1999 and version 10 from 2000/1 onwards) were used to identify causes of death. Birthweights were classified into four groups: 500-1,499g (VLBW), 1,500-2499g (LBW), 2,500-3499g and ≥3,500g. The latter two groups were merged for some analyses. The index of multiple deprivation (IMD), which is based on a combined measure of deprivation including wealth, schooling, home ownership in a specific area,* *was divided into quintiles separately for England and Wales then combined into a single variable. Although there were few missing values for the covariates (maximum of 22,061 (0.2%) for IMD), these were unlikely to influence the overall conclusions due to the large dataset.****”*** |
| Bias | | 9 | Describe any efforts to address potential sources of bias  *Efforts to address potential sources of bias are described in the methods section fifth paragraph of the main manuscript*  *“Several sensitivity analyses were conducted. Since congenital malformations are associated with increased mortality, the analyses were repeated after exclusion of deaths from congenital malformations. Due to overall decrease in mortality over the last two decades, that could influence the results, we confined the analyses to the most recent 5-year period. In addition, deaths were classified according to the age bands 1-5, 6-10 and 11-18 years of age. Finally, to assess the potential role of gestation, we repeated the analyses for Welsh data as gestation was available for all births and deaths for the study period. For Welsh data, we also compared the infant mortality rates between those who had intrauterine growth restriction (IUGR, <10% centile for birthweight adjusted for gender and gestation) program with appropriate birthweight for their gestation (AGA, 20 – 80% centile) using the LMS Growth (Medical Research Council, UK) .”* |
| Study size | | 10 | Explain how the study size was arrived at  *How the study size was arrived at is describe in the methods section first paragraph of the main manuscript*  *“Anonymised data for all live-births and deaths up to 18 years of age occurring in England and Wales between 1993 and 2011 were available (Office for National Statistics, ONS). The data included birthweight (babies are routinely weighed shortly after birth on calibrated scales) and the covariates gender, local deprivation score, maternal age and maternal parity as these have been shown to be associated with increased mortality . In addition, the data included age and cause of each death. ONS data for infant deaths were provided already classified into early, late and post-neonatal deaths; beyond infancy, age in completed years at time of death were provided. Gestational age at birth is not routinely recorded for the deaths except for the Welsh data. Welsh data, was obtained from the All Wales Perinatal Survey (*[*https://awpsonline.uk/*](https://awpsonline.uk/)*), as a separate cohort for the same time period. International Classification of Diseases (ICD) code (version 9 up to 1999 and version 10 from 2000/1 onwards) were used to identify causes of death. Birthweights were classified into four groups: 500-1,499g (VLBW), 1,500-2499g (LBW), 2,500-3499g and ≥3,500g. The latter two groups were merged for some analyses. The index of multiple deprivation (IMD), which is based on a combined measure of deprivation including wealth, schooling, home ownership in a specific area,* *was divided into quintiles separately for England and Wales then combined into a single variable. Although there were few missing values for the covariates (maximum of 22,061 (0.2%) for IMD), these were unlikely to influence the overall conclusions due to the large dataset.”* |
| Quantitative variables | | 11 | Explain how quantitative variables were handled in the analyses. If applicable, describe which groupings were chosen and why  How quantitative variables were handled in the analyses is described in the methods section first paragraph in the main manuscript.  “Birthweights were classified into four groups: 500-1,499g (VLBW), 1,500-2499g (LBW), 2,500-3499g and ≥3,500g. The latter two groups were merged for some analyses. The index of multiple deprivation (IMD), which is based on a combined measure of deprivation including wealth, schooling, home ownership in a specific area, was divided into quintiles separately for England and Wales then combined into a single variable.” |
| Statistical methods | | 12 | (*a*) Describe all statistical methods, including those used to control for confounding |
|  |  |  | (*b*) Describe any methods used to examine subgroups and interactions |
|  |  |  | (*c*) Explain how missing data were addressed |
|  |  |  | (*d*) *Cohort study*—If applicable, explain how loss to follow-up was addressed  *Case-control study*—If applicable, explain how matching of cases and controls was addressed  *Cross-sectional study*—If applicable, describe analytical methods taking account of sampling strategy |
|  |  |  | (*e*) Describe any sensitivity analyses  *The statistical methods are given in the methods paragraphs 2-5 of the main manuscript.*  *“Cox proportional hazards regression was specified as a means of analysing this data through estimation of hazard ratios and their associated 95% confidence intervals for the birthweight groups for infant mortality (death up to 12 months of age) and mortality between 1 and 18 years of age, after censoring deaths occurring in infancy. A Cox model was used to quantify differences between survival rates for the birthweight groups with and without adjustments for relevant covariates. The proportional hazard assumption for the birthweight groups was tested in each model by the addition of an appropriate time-dependant covariate – a product of the system time variable called T_ and the variable age. All covariates were categorical to permit possible non-linear responses. Mortality rates are also given in person years.*  *Inspired by peer review, we additionally considered how utilisation of birthweight as a continuous variable might change the results. We used Welsh infant mortality data, where continuous birthweight was expressed as a 5 knot restricted cubic spline to provide a linear component within the Cox regression . The knots were chosen in terms of birthweight at 0.5, 1.5, 2.5, 3.5 and 4.5 kg to approximately mimic the categorical birthweight bandings. A 5 knot spline has X (birthweight) and X2, X3 and X4 which are all functions in X. The hazard function within the survival model is then*  *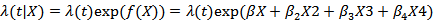*  *Also following peer recommendation, we utilised the completeness of the data up to age 10 years of age i.e. every child in the cohort would have either turned 10 or died, to calculate the Population Attributable Fraction (PAF) for deaths from 1 to 10 year olds .*  *Several sensitivity analyses were conducted. Since congenital malformations are associated with increased mortality, the analyses were repeated after exclusion of deaths from congenital malformations. Due to overall decrease in mortality over the last two decades, that could influence the results, we confined the analyses to the most recent 5-year period. In addition, deaths were classified according to the age bands 1-5, 6-10 and 11-18 years of age. Finally, to assess the potential role of gestation, we repeated the analyses for Welsh data as gestation was available for all births and deaths for the study period. For Welsh data, we also compared the infant mortality rates between those who had intrauterine growth restriction (IUGR, <10% centile for birthweight adjusted for gender and gestation) program with appropriate birthweight for their gestation (AGA, 20 – 80% centile) using the LMS Growth (Medical Research Council, UK) .”* |
| Results | | | |
| Participants | 13* | (a) Report numbers of individuals at each stage of study—eg numbers potentially eligible, examined for eligibility, confirmed eligible, included in the study, completing follow-up, and analysed | |
|  |  | (b) Give reasons for non-participation at each stage | |
|  |  | (c) Consider use of a flow diagram  *Information on participants is given in the results first paragraph and table 1 of the main manuscript.*  ***“Results***  ***All-Cause Mortality***  *The total cohort included 12,457,528 live-births born in England and Wales between 1993 and 2011. Birthweight data were missing for 92,456 cases and data for 9,821 cases were considered implausible or were outside the limits of analyses including birthweight of <500g. The characteristics of the included 12,355,251 cases representing 121,208,305 person years are shown in Table 1. There were 139,608 (1.1% of all live-births), 759,283 (6.1%), 6,511,411 (52.7%), and 4,944,949 (40.0%) live-births in the 500-1,499g, 1,500-2,499g, 2,500-3,499g and ≥3,500g groups respectively. In total, 74,890 (0.61%) of live-births died between birth and 18 years of age with 77% of deaths occurring in the first 12 months of life and 23% between 1 and 18 years of age. The mortality rate per 100,000 person years was 466 for infant deaths and 15.9 for children aged between 1 and 18 years. For the four birthweight groups there were 25,414 (18.20%), 11,945 (1.57%), 25,750 (0.40%) and 11,781 (0.24%) deaths respectively.****”*** | |
| Descriptive data | 14* | (a) Give characteristics of study participants (eg demographic, clinical, social) and information on exposures and potential confounders | |
|  |  | (b) Indicate number of participants with missing data for each variable of interest | |
|  |  | (c) *Cohort study*—Summarise follow-up time (eg, average and total amount)  *Descriptive data is given in the results first paragraph of the main manuscript.*  ***“Results***  ***All-Cause Mortality***  *The total cohort included 12,457,528 live-births born in England and Wales between 1993 and 2011. Birthweight data were missing for 92,456 cases and data for 9,821 cases were considered implausible or were outside the limits of analyses including birthweight of <500g. The characteristics of the included 12,355,251 cases representing 121,208,305 person years are shown in Table 1. There were 139,608 (1.1% of all live-births), 759,283 (6.1%), 6,511,411 (52.7%), and 4,944,949 (40.0%) live-births in the 500-1,499g, 1,500-2,499g, 2,500-3,499g and ≥3,500g groups respectively. In total, 74,890 (0.61%) of live-births died between birth and 18 years of age with 77% of deaths occurring in the first 12 months of life and 23% between 1 and 18 years of age. The mortality rate per 100,000 person years was 466 for infant deaths and 15.9 for children aged between 1 and 18 years. For the four birthweight groups there were 25,414 (18.20%), 11,945 (1.57%), 25,750 (0.40%) and 11,781 (0.24%) deaths respectively.”* | |
| Outcome data | 15* | *Cohort study*—Report numbers of outcome events or summary measures over time  *Numbers of outcome events were reported in the first paragraph of the results section of the main manuscript and table 1.*  ***“Results***  ***All-Cause Mortality***  *The total cohort included 12,457,528 live-births born in England and Wales between 1993 and 2011. Birthweight data were missing for 92,456 cases and data for 9,821 cases were considered implausible or were outside the limits of analyses including birthweight of <500g. The characteristics of the included 12,355,251 cases representing 121,208,305 person years are shown in Table 1. There were 139,608 (1.1% of all live-births), 759,283 (6.1%), 6,511,411 (52.7%), and 4,944,949 (40.0%) live-births in the 500-1,499g, 1,500-2,499g, 2,500-3,499g and ≥3,500g groups respectively. In total, 74,890 (0.61%) of live-births died between birth and 18 years of age with 77% of deaths occurring in the first 12 months of life and 23% between 1 and 18 years of age. The mortality rate per 100,000 person years was 466 for infant deaths and 15.9 for children aged between 1 and 18 years. For the four birthweight groups there were 25,414 (18.20%), 11,945 (1.57%), 25,750 (0.40%) and 11,781 (0.24%) deaths respectively.”* | |
|  |  | *Case-control study—*Report numbers in each exposure category, or summary measures of exposure | |
|  |  | *Cross-sectional study—*Report numbers of outcome events or summary measures | |
| Main results | 16 | (*a*) Give unadjusted estimates and, if applicable, confounder-adjusted estimates and their precision (eg, 95% confidence interval). Make clear which confounders were adjusted for and why they were included*.*  *Table 1, 2 and Fig 1,2 of the main manuscript and tables S9, S10 and S3 Fig of the online supplement and paragraphs 2-4 in the results section in the main manuscript provides the above data.*  *“Infant mortality rates were significantly greater in the lower birthweight groups: the rate ratio and rates per 100,000 person years were 129.7 and 1,985.2 for the 500-1,499g; 9.5 and 145.3 for the 1,500-2,499g; 1.9 and 28.8 for the 2,500-3,499g and 1.0 and 15.3 for the ≥3,500g groups respectively. In addition, boys fared worse than girls (S1 Fig and S2 Fig) even with inclusion and exclusion of external factors as defined by ICD codes relative risk female:male deaths were 0.79 and 0.78 with and without external factors respectively for the <1 year group. For the 1-18 years of age group relative risk female:male deaths were 0.82 and 0.87 with and without external factors respectively. Increased deprivation and multiple births were associated with greater deaths for all birthweight groups. Maternal age showed a U-shaped relationship with greatest mortality in the younger and older mothers (Table 1). The Population Attributable Fraction (PAF) for infant deaths and for deaths between 1 and 10 years of age were 56.6% and 10.7% respectively for the comparison between the <2,500g group and ≥2,500g groups; and 51.0% and 3.8% respectively when the <1,500g group and ≥2,500g groups were compared.*  *For deaths occurring between 1 and 18 years of age, the rates per 100,000 person years were 53.2, 29.5, 14.4 and 10.6 respectively for the 500-1,499g, 1,500-2,499g, 2,500-2,499g and the ≥3,500g groups. The rate ratios were 5.0, 2.8, and 1.3 compared to the ≥3,500g group. Gender, deprivation and maternal age bands showed similar patterns as for infant deaths, but the difference between singletons and multiple births was decreased.*  *Fig 1 and Fig 2 show survival curves for the four birthweight groups in infancy and between 1 and 18 years of age (after censoring deaths occurring in infancy). Whilst confirming the association of low birthweight and increased mortality in infancy, the mortality rates were also increased for the lower birthweight groups in childhood/adolescence. Table 2 shows hazard ratios adjusted for deprivation alone and for deprivation, maternal age, multiple birth status and gender. An inverse relationship was noted between the birthweight groups and mortality which was largely unaffected after adjustments for covariates.*  *When Welsh infant mortality data was investigated utilising birthweight splines, we noted the spline function:*  *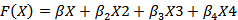*  *The coefficients 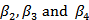 where not significant (S9 Table in the supplementary information) which implies that the log of the hazard function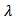 is basically linear in birth weight (see S3 Fig in the supplementary information). The actual expression is*  *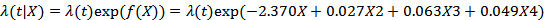*  *When the Hazard ratios were calculated at the mid points between the knots, the values were very close to those noted following Cox regression of the Welsh data using the categorical definitions (see S10 Table in the supplementary information). ”* | |
|  |  | (*b*) Report category boundaries when continuous variables were categorized  *Table 1, 2 and Fig 1,2 of the main manuscript and tables S9, S10 and S3 Fig of the online supplement and paragraphs 2-4 in the results section in the main manuscript provides the above data.*  *“Infant mortality rates were significantly greater in the lower birthweight groups: the rate ratio and rates per 100,000 person years were 129.7 and 1,985.2 for the 500-1,499g; 9.5 and 145.3 for the 1,500-2,499g; 1.9 and 28.8 for the 2,500-3,499g and 1.0 and 15.3 for the ≥3,500g groups respectively. In addition, boys fared worse than girls (S1 Fig and S2 Fig) even with inclusion and exclusion of external factors as defined by ICD codes relative risk female:male deaths were 0.79 and 0.78 with and without external factors respectively for the <1 year group. For the 1-18 years of age group relative risk female:male deaths were 0.82 and 0.87 with and without external factors respectively. Increased deprivation and multiple births were associated with greater deaths for all birthweight groups. Maternal age showed a U-shaped relationship with greatest mortality in the younger and older mothers (Table 1). The Population Attributable Fraction (PAF) for infant deaths and for deaths between 1 and 10 years of age were 56.6% and 10.7% respectively for the comparison between the <2,500g group and ≥2,500g groups; and 51.0% and 3.8% respectively when the <1,500g group and ≥2,500g groups were compared.*  *For deaths occurring between 1 and 18 years of age, the rates per 100,000 person years were 53.2, 29.5, 14.4 and 10.6 respectively for the 500-1,499g, 1,500-2,499g, 2,500-2,499g and the ≥3,500g groups. The rate ratios were 5.0, 2.8, and 1.3 compared to the ≥3,500g group. Gender, deprivation and maternal age bands showed similar patterns as for infant deaths, but the difference between singletons and multiple births was decreased.*  *Fig 1 and Fig 2 show survival curves for the four birthweight groups in infancy and between 1 and 18 years of age (after censoring deaths occurring in infancy). Whilst confirming the association of low birthweight and increased mortality in infancy, the mortality rates were also increased for the lower birthweight groups in childhood/adolescence. Table 2 shows hazard ratios adjusted for deprivation alone and for deprivation, maternal age, multiple birth status and gender. An inverse relationship was noted between the birthweight groups and mortality which was largely unaffected after adjustments for covariates.*  *When Welsh infant mortality data was investigated utilising birthweight splines, we noted the spline function:*  *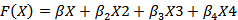*  *The coefficients 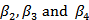 where not significant (S9 Table in the supplementary information) which implies that the log of the hazard function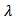 is basically linear in birth weight (see S3 Fig in the supplementary information). The actual expression is*  *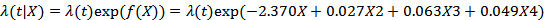*  *When the Hazard ratios were calculated at the mid points between the knots, the values were very close to those noted following Cox regression of the Welsh data using the categorical definitions (see S10 Table in the supplementary information). ”* | |
|  |  | (*c*) If relevant, consider translating estimates of relative risk into absolute risk for a meaningful time period | |
| Other analyses | 17 | Report other analyses done—eg analyses of subgroups and interactions, and sensitivity analyses  *The sensitivity analysis is reported in the final three paragraphs of the results section of the main manuscript with the tables in the supporting information file.*  ***“Sensitivity Analyses***  *Congenital Malformations*  *Results were only slightly different when congenital malformations were removed (supporting information file S1 Table and S2 Table).*  *Gestational Age*  *Gestational age was available for all Welsh live-births via the All Wales Perinatal Survey (*[*https://awpsonline.uk/*](https://awpsonline.uk/)*). For the study period, there were 635,428 live-births and 3,836 deaths between birth and 18 years of age for birthweights above 500g. For VLBW, the median gestation was 29 weeks with 10^th^ and 90^th^ percentiles at 25 and 33 weeks respectively. Corresponding data were 36 (32, 39), 39 (37, 41) and 40 (39, 41) weeks for the LBW, 2500-3,499g and ≥3,500g groups respectively. Supporting information file S3 Table shows the Cox regression results for the two age groups and were very similar to the overall results. For all gestation groups, there was an increased risk of death in infancy if the infant had IUGR when compared to appropriately grown infants (S4 Table) although the difference was not significant for the 23 – 28 weeks’ gestation group.*  *Temporal effects on mortality*  *The time varying covariate was significant in all regression models suggesting that hazard ratios may be temporally affected. The effect of adjustment for the time varying covariate only slightly increased the hazard ratios (supporting information file S5 Table). When the data were classified into shorter time-periods (1993-1996, 1997-2001, 2002-2006 and 2007-2011) the results remained essentially unchanged with small increases for each time period (supporting information file S6 Table and S7 Table). When deaths up to 18 years were divided into shorter age groups, younger groups had greater mortality (hazard ratio of 8.0 for 1-5 year-olds, 4.2 for 6-10 year-olds and 2.7 for 11-18 year-olds) (supporting information file S8 Table).”* | |
| Discussion | | | |
| Key results | 18 | Summarise key results with reference to study objectives  *The key results stated in the first paragraph of the discussion of the main manuscript.*  *“****Discussion***  *This population-based study included data from 12,457,528 live-births occurring in England and Wales between 1993 and 2011. Overall 0.61% (n=74,890) died with 23% of all deaths occurring after infancy up to 18 years of age. Mortality was greatest in the lower birthweight groups in infancy and between 1-18 years of age with little modification by covariates. Adjusted hazard ratios for deaths in infancy were 145 (141,149) and 9.8 (9.5, 10.1) for VLBW and LBW groups respectively when compared to the ≥3,500g group and 6.6 (6.1, 7.1) and 2.9 (2.8, 3.1) for these birthweight groups between 1-18 years of age. Gender, maternal age, multiple births and deprivation also contributed to increased deaths in the lower birthweight groups. Perinatal factors and congenital malformations especially from cardiovascular causes were predominant in the lower birthweight groups in infancy and later in childhood and adolescence.”* | |
| Limitations | 19 | Discuss limitations of the study, taking into account sources of potential bias or imprecision. Discuss both direction and magnitude of any potential bias  *The limitations and implications are discussed in the ninth paragraph of the discussion of the main manuscript*  *“We had little missing data but we did not have gestational age nor maternal smoking. Since the former is highly correlated with birthweight and the latter with deprivation, we believe that our findings remain robust despite these short-comings. Additionally, by using birthweight as a continuous variable for the Welsh data, we were able to show that there was no disadvantage in using banded as opposed to continuous birthweight data.”* | |
| Interpretation | 20 | Give a cautious overall interpretation of results considering objectives, limitations, multiplicity of analyses, results from similar studies, and other relevant evidence  *The interpretation is discussed in the tenth paragraph of the discussion of the main manuscript.*  *“In conclusion, using what we believe to be the largest and most recent population-based cohort which has been used to investigate this area, we have shown that VLBW and LBW are associated with mortality in infancy and in childhood/adolescence. Major causes include perinatal and congenital malformations in infancy but continue to explain many deaths that occur up to 18-years of age. Low birthweight is clearly associated with later mortality including in childhood and adolescence. By understanding and ameliorating influences of upstream exposures such maternal smoking and deprivation, later mortality can be decreased by reducing the delivery of vulnerable infants with low birthweight.”* | |
| Generalisability | 21 | Discuss the generalisability (external validity) of the study results  *The generalisability is discussed in the tenth paragraph of the discussion of the main manuscript.*  *“In conclusion, using what we believe to be the largest and most recent population-based cohort which has been used to investigate this area, we have shown that VLBW and LBW are associated with mortality in infancy and in childhood/adolescence. Major causes include perinatal and congenital malformations in infancy but continue to explain many deaths that occur upto 18-years of age. Low birthweight is clearly associated with later mortality including in childhood and adolescence. By understanding and ameliorating influences of upstream exposures such maternal smoking and deprivation, later mortality can be decreased by reducing the delivery of vulnerable infants with low birthweight.”* | |
| Other information | | | |
| Funding | 22 | Give the source of funding and the role of the funders for the present study and, if applicable, for the original study on which the present article is based  *As per PLOS medicines guidance we have not included funding sources in the Acknowledgments or anywhere else in the manuscript file. Funding information will be entered in the financial disclosure section of the online submission system. As follows*  *“The study was funded by the Welsh Government (All Wales Perinatal Survey) and Cardiff University is the employer of all the authors.”* | |

*Give information separately for cases and controls in case-control studies and, if applicable, for exposed and unexposed groups in cohort and cross-sectional studies.

**Note:** An Explanation and Elaboration article discusses each checklist item and gives methodological background and published examples of transparent reporting. The STROBE checklist is best used in conjunction with this article (freely available on the Web sites of PLoS Medicine at http://www.plosmedicine.org/, Annals of Internal Medicine at http://www.annals.org/, and Epidemiology at http://www.epidem.com/). Information on the STROBE Initiative is available at www.strobe-statement.org.
